# Optimizing the Relaxivity of MRI Probes at High Magnetic Field Strengths With Binuclear Gd^III^ Complexes

**DOI:** 10.3389/fchem.2018.00158

**Published:** 2018-05-15

**Authors:** Loredana Leone, Giuseppe Ferrauto, Maurizio Cossi, Mauro Botta, Lorenzo Tei

**Affiliations:** ^1^Dipartimento di Scienze e Innovazione Tecnologica, Università degli Studi del Piemonte Orientale “Amedeo Avogadro”, Alessandria, Italy; ^2^Department of Molecular Biotechnology and Health Sciences, Molecular Imaging Centre, University of Torino, Torino, Italy

**Keywords:** gadolinium, macrocyclic ligands, multimeric contrast agents, magnetic resonance imaging-high field, chemical exchange saturation transfer, relaxometry, DFT

## Abstract

The key criteria to optimize the relaxivity of a Gd(III) contrast agent at high fields (defined as the region ≥ 1.5 T) can be summarized as follows: (i) the occurrence of a rotational correlation time τ_R_ in the range of ca. 0.2–0.5 ns; (ii) the rate of water exchange is not critical, but a τ_M_ < 100 ns is preferred; (iii) a relevant contribution from water molecules in the second sphere of hydration. In addition, the use of macrocycle-based systems ensures the formation of thermodynamically and kinetically stable Gd(III) complexes. Binuclear Gd(III) complexes could potentially meet these requirements. Their efficiency depends primarily on the degree of flexibility of the linker connecting the two monomeric units, the absence of local motions and the presence of contribution from the second sphere water molecules. With the aim to maximize relaxivity (per Gd) over a wide range of magnetic field strengths, two binuclear Gd(III) chelates derived from the well-known macrocyclic systems DOTA-monopropionamide and HPDO3A (Gd_2_**L1** and Gd_2_**L2**, respectively) were synthesized through a multistep synthesis. Chemical Exchange Saturation Transfer (CEST) experiments carried out on Eu_2_**L2** at different pH showed the occurrence of a CEST effect at acidic pH that disappears at neutral pH, associated with the deprotonation of the hydroxyl groups. Then, a complete ^1^H and ^17^O NMR relaxometric study was carried out in order to evaluate the parameters that govern the relaxivity associated with these complexes. The relaxivities of Gd_2_**L1** and Gd_2_**L2** (20 MHz, 298 K) are 8.7 and 9.5 mM^−1^ s^−1^, respectively, +77% and +106% higher than the relaxivity values of the corresponding mononuclear GdDOTAMAP-En and GdHPDO3A complexes. A significant contribution of second sphere water molecules was accounted for the strong relaxivity enhancement of Gd_2_**L2**. MR phantom images of the dinuclear complexes compared to GdHPDO3A, recorded at 7 T, confirmed the superiority of Gd_2_**L2**. Finally, ab initio (DFT) calculations were performed to obtain information about the solution structure of the dinuclear complexes.

## Introduction

The success of Magnetic Resonance Imaging (MRI) as a clinical diagnostic technique is mainly related to its superb temporal and spatial resolution that allow the clear delineation and differentiation of soft tissues and to its low invasiveness that leads to high patient acceptability. MRI contrast agents (CAs) are used for a large fraction of clinical scans (40–50%) to increase tissue contrast on relaxation weighted images by shortening the relaxation times of the water molecules in their proximity. Some 400 million doses of gadolinium have been administered for MRI scans since 1988. Currently, clinically employed CAs are low molecular weight, nonspecific Gd^III^ complexes with polyaminocarboxylate ligands that are capable to enhance the longitudinal relaxation rate (*R*_1_ = 1/*T*_1_) of water protons in the extracellular space. The increase in *R*_1_ induced by one millimolar concentration of the paramagnetic ion is called relaxivity (*r*_1_), a key parameter that depends on several structural and dynamic features of the Gd^III^ complex. Among the most important are the molecular rotation (τ_R_), the electronic relaxation times (*T*_1, 2e_) and the residence lifetime of the coordinated water molecule(s) (τ_M_) (Caravan et al., 1999; Botta and Tei, 2012; Merbach et al., 2013).

Typically, the commercially available Gd-based CAs have limited contrast enhancement capability (*r*_1_ about 3–4 mM^−1^s^−1^ at 0.47 T and 37°C), much lower than that theoretically attainable (Geraldes and Laurent, 2009). Furthermore, their relaxivity values steadily decrease with increasing the magnetic field strength, e.g., *r*_1_ of GdHPDO3A at 11.7 T and 37°C is 2.9 mM^−1^s^−1^ (Delli Castelli et al., 2013). Therefore, over the years, great research efforts have been made to optimize the structural and dynamic properties of the Gd^III^ complexes in order to achieve higher relaxivities, in particular in the high fields range. However, the interplay of the different contributions often resulted in non-optimized systems or probes that afford remarkable results only for a specific application. For example, blood pool contrast agents, such as MS-325, were designed to exploit their strong binding to slow tumbling molecules (i.e., Human Serum Albumin) for MR angiographic applications (Caravan et al., 2002), but this strategy gives high longitudinal relaxivity between 0.5 and 1.0 T and then sharply drops with increasing field thus, at fields > 1.5 T, macromolecular agents are hardly superior to small molecular weight chelates.

Nonetheless, although nowadays the majority of MRI scanners used in clinics operates at 1.5 T, more than one-quarter are 3 T machines and 7 T whole body human scanners are also available on the market. Therefore, a specific strategy for *r*_1_ enhancement of metal-based probes at high field, i.e., a Gd-complex that will maintain its *r*_1_ constant at least in the 1.5–3.0 T range, is extremely necessary to obtain a better contrast enhancement with lower amounts of CAs administered.

In recent reviews, it has been nicely shown that the use of systems with rotational correlation times in the range of ~0.5–2.0 ns and coordinated water molecule(s) in a relatively fast exchange rate (τ_M_ ~10–100 ns) would allow the increase of *r*_1_ at magnetic field strengths > 1.5 T (Caravan et al., 2009; Helm, 2010). However, in accordance with recent investigations, we surmised that ditopic Gd^III^ chelates with a molecular rotational correlation time (τ_R_) in the range 0.2–0.5 ns (i.e., Gd-complexes with molecular mass in the range of ca. 1–3 kDa) and a short, rigid and hydrophilic linker between the two chelating units could represent an optimal solution. In fact, in such systems the rigidity of the spacer should reduce the local mobility of the monomeric Gd-units and enable a better correlation between global and local motions. Moreover, a hydrophilic spacer might favor the presence of a network of hydrogen bonded water molecules that can contribute greatly to the relaxivity through the second sphere contribution (Botta, 2000). A residence lifetime of the inner sphere water molecule (τ_M_) preferably below 100 ns would complete the characteristics of these ditopic Gd^III^ chelates.

**Scheme 1:**
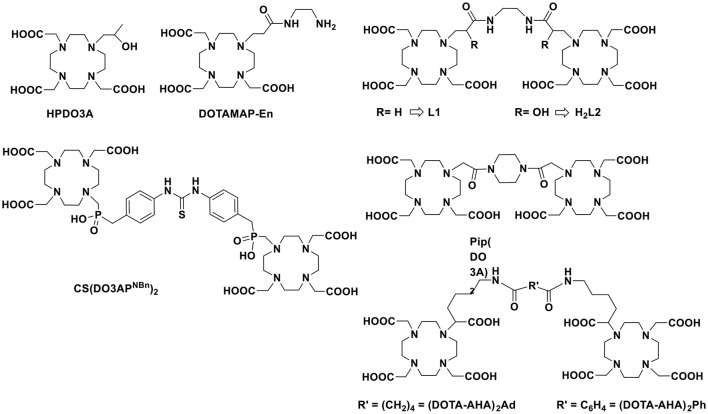
Chemical structure of chelating ligands discussed in this work.

There are several approaches to design ditopic Gd-complexes as clearly delineated by Caravan and co-workers (Boros et al., 2012), being the one with an organic scaffold at the barycentre the most used strategy. In their example, they used an amino acid-like, fast exchanging Gd-complex (DOTAla) suitable for solid phase peptide synthesis and integration into hydrophilic polypeptide structures. They also investigated the possibility to rigidify the multimeric structure by forming disulphide bridges between cysteine moieties. Other examples of ditopic systems were reported by using GdDOTA-monoamide (Powell et al., 1996; Tei et al., 2009b), GdHPDO3A (Ranganathan et al., 1998), GdDOTA (Fontes et al., 2015) or GdDOTA-monophosphinate (Rudovský et al., 2006) derivatives linked by aliphatic or heterocyclic groups (Scheme 1). The relaxivity enhancement of these systems as compared to monomeric Gd-complexes was often very much dependent on the water exchange rate or on the rigidity of the linker. We were also inspired by the recently reported self-assembling of two oppositely charged macrocyclic Gd-chelates that resulted in a small-sized dimeric system with high relaxivity at high frequencies thanks to improved inner- and second-sphere relaxivity contributions (Lawson et al., 2015). Remarkably, the additional contribution of about 30–40% from water molecules in the second coordination sphere was considered responsible of the increased performance of the ditopic probe.

Finally, we must consider that the kinetic inertness toward dissociation/transmetallation reactions is a fundamental parameter to consider in the design of novel MRI probe to aim for *in vivo* applications. Therefore, although a couple of examples of kinetically inert *q* = 2 Gd-complexes have been reported (Tircsó et al., 2006; Vagner et al., 2016), we will concentrate on two well-established *q* = 1 systems based on the macrocyclic systems HPDO3A (10-(2-hydroxypropyl)-1,4,7-tetraazacyclododecane-1,4,7-triacetic acid) and DOTAMAP (1,4,7,10-tetraazacyclododecane-1-propionamido-4,7,10-triacetic acid). These two chelates have been chosen because GdHPDO3A is the well-established, clinically approved MRI agent ProHance commercialized by Bracco (Delli Castelli et al., 2013) and GdDOTAMAP has been reported as a kinetically inert and fast exchanging Gd-complex (Tei et al., 2009a, 2015) that has also been recently exploited for the synthesis of high relaxivity multimeric systems (Boros et al., 2012). Thus, we present here the synthesis of two new ditopic chelators **L1** (DOTAMAP)_2_ and **H**_2_**L2** (HPA-DO3A)_2_ (Scheme 1) and a complete ^1^H and ^17^O NMR relaxometric study on the Gd^III^ complexes and a CEST characterization on Eu_2_**L2**. Furthermore, ab initio DFT calculations were used to obtain information on the structure of Gd_2_**L2** in solution, on the possible presence of second hydration sphere water molecules and on the internal mobility of the chelates about the linker.

## Materials and methods

All chemicals were purchased from Sigma-Aldrich or Alfa Aesar unless otherwise stated and were used without further purification. The ^1^H and ^13^C NMR spectra were recorded using a Bruker Advance III 500 MHz (11.4 T) spectrometer equipped with 5mm PABBO probes and BVT-3000 temperature control unit. Chemical shifts are reported relative to TMS and were referenced using the residual proton solvent resonances. HPLC analyses and mass spectra were performed on a Waters HPLC-MS system equipped with a Waters 1525 binary pumps. Analytical measurements were carried out on a Waters Atlantis RPC18 column (5 μm 4.6 × 100 mm) and on a Waters Atlantis prep T3 OBD (5 μm 19 × 100 mm) for preparative purposes. Electrospray ionization mass spectra (ESI MS) were recorded using a SQD 3100 Mass Detector (Waters), operating in positive or negative ion mode, with 1% v/v formic acid in methanol as the carrier solvent.

### 1,2-diacrylamidoethane

To a stirred solution of ethylenediamine (0.110 mL, 100 mg, 1.66 mmol) and K_2_CO_3_ (0.7 g, 5 mmol) in dry CH_3_CN (2 mL), acryloyl chloride (0.3 mL, 0.33 g, 3.65 mmol) was added dropwise at 0°C and left stirring at r.t overnight, then filtered and evaporated in vacuo to obtain the pure product. Analytical HPLC: Pump A: NH_4_OAc (7 mM, pH 5.5); Pump B = ACN; flow = 1 mL/min; 0–10 min = 100% A; 10–15 min = 100% B; 15–24 min = 100% B; 24–25 min = 100% A; t_r_ = 17.18 min.

Yield 250 mg (90%); MS (ESI^+^): *m/z*: 169.17 [M+H]^+^^1^H NMR (500MHz, CD_3_CN, 25°C): δ = 6.85 (brs, 1H; NH-), 6.14 (m, ^2^J (H,H) = 2.5 Hz, ^3^J (H,H) = 4.8 Hz, 2H; C*H*_2_ = CH), 5.59 (dd, ^2^J (H,H) = 2.5 Hz, ^3^J (H,H) = 4.8 Hz, 1H; CH_2_ = *CH*-), 3.33 (d, ^3^J (H,H) = 2.8 Hz; 2H, CO-NH-C*H*_2_) ppm. ^13^C{1H}NMR (125MHz, CD_3_CN, 25°C): δ = 166.6 (*CO-*NH-CH_2_), 132.4 (CH_2_ = *C*H), 125.9 (*C*H_2_ = CH), 39.9 (-CO-NH-*C*H_2_-) ppm.

### Gd_2_(DOTAMAP)_2_ (Gd_2_L1)

A solution of DO3A(t-Bu)_3_ (50 mg, 0.097 mmol) and DIPEA (30 μL, 0.17 mmol) in DMF (2.5 mL) was added dropwise to 1,2-diacrylamidoethane (7 mg, 0.044 mmol) in DMF (1 mL) under N_2_. The reaction was allowed to stir at 80°C overnight. Then, the crude mixture was evaporated in vacuo and the resulting oil was dissolved in a solution 1:1 of DCM: TFA and stirred at room temperature overnight. Then, the solvent mixture was evaporated under reduced pressure to obtain a yellow oil that was dissolved in H_2_O. Complexation was performed directly on the unpurified sample: Ln(NO_3_)_3_ (Ln = Gd and Eu; 0.5 M aqueous solutions) was added to a solution of the ligand in water (2 mL). The pH was gradually adjusted to 7 and the solution stirred at room temperature overnight. The pH was then increased to 10 to precipitate excess Ln^III^ as hydroxide. The solution was centrifuged (4,000 rpm, 3 min, r.t.) and the supernatant filtered through a 0.2 μm filter. The pH was re-adjusted to 7 and the solvent removed in vacuo. The Ln^III^-complexes were purified on preparative HPLC with the following method: Pump A: NH_4_OAc (7 mM, pH 5.5); Pump B = ACN; flow = 20 mL/min; 0–4 min = 100% A; 4–14 min = 100% B; 14–15 min = 100% B; 15–16 min = 100% A. Gd_2_L1: t_r_ = 6.69 min; Yield: 30% (16 mg, 0.014 mmol). MS (ESI^+^): *m/z*: 585.2 [(M+2H)/2]^+^. Eu_2_**L1**: t_r_ = 7.01 min Yield: 25% (12 mg, 0.010 mmol). MS (ESI^+^): *m/z*: 580.1 [(M+2H)/2]^+^.

### *N*,*N*′-(ethane-1,2-diyl)bis(oxirane-2-carboxamide)

1,2-diacrylamidoethane (100 mg, 0.6 mmol) and *m*-chloroperbenzoic acid (1 g, 4.76 mmol) were dissolved in CH_3_CN (3 mL) and reacted in a MW (CEM, DISCOVER SP) reactor (45 min, 95°C, 200 W). The solution was then evaporated in vacuo, dissolved in 1 mL H_2_O:ACN (1:1) and purified in preparative HPLC with the following method: Pump A:NH_4_OAc (7 mM, pH 5.5); Pump B = ACN; flow = 20 mL/min; 0–10 min = 100% A; 10–15 min = 100% B; 15–24 min = 100% B; 24–25 min = 100% A; t_r_ = 13.06 min. Forty-eight milligram (0.24 mmol, 40% yield) of a white powder were obtained. MS (ESI^+^): *m/z*: 201.25 [M+H]^+^; ^1^H NMR (500MHz, CD_3_CN, 25°C): δ = 3.27 (m, 2H; CO-NH-C*H*_2_,), 3.20 (m, 1H; CO-C*H-*CH2), 2.89, 2.73 (m,m, 2H; CO-CH-C*H*_2_) ppm. ^13^C{1H}NMR (125 MHz, CD_3_CN, 25°C): δ = 170.6 (*CO-*NH-CH_2_), 49.9 (CO-CH-*CH*_2_) 47.6 (CO-*CH*-CH_2_), 39.6 (CO-NH-*CH*_2_) ppm.

### (HPA-DO3A)_2_(*t*Bu)_6_

*N*,*N*′-(ethane-1,2-diyl)bis(oxirane-2-carboxamide) (18 mg, 0.09 mmol) was dissolved in dry CH_3_CN (5 mL) and K_2_CO_3_ (50 mg, 0.36 mmol) and DO3A(*t*-Bu)_3_ (92 mg, 0.18 mmol) were added to the solution and stirred overnight at room temperature. After filtration and evaporation under reduced pressure, the mixture was purified on silica (CH_3_CN: NH_3_ (10%)) to give the product **2**. Analytical HPLC: Pump A: H_2_O (0.1% TFA); Pump B = MeOH; flow = 1 mL/min; 0-3 min = 70% A; 3-18 min = 100% B; 18-24 min = 100% B; 24-25 min = 70% A; t_r_ = 14.58 min. 28 mg (0.023 mmol, 25% yield) of a pale yellow oil were obtained. MS (ESI^+^): *m/z*: 615.8 [(M+2H)/2]^+^. ^1^H NMR (500MHz, CD_3_CN, 25°C): δ = 3.89 (dd, 2H; N-CH_2_-C*H-*CO-), 3.29 (m, 4H; N-C*H*_2_ -CH-CO), 3.20 (m,12 H; N-C*H*_2_-CO-OC(CH_3_)_3_), 2.68-2.84 (m, 32H; cyclen), 1.44 (s, 54 H; N-CH_2_-CO-O*C(CH*_3_*)*_3_) ppm. ^13^C{^1^H}NMR (125MHz, CD_3_CN, 25°C): δ = 174.8 (N-CH_2_-*CO-*OC(CH_3_)_3_), 172.0 (N-CH_2_-CH-*CO*); 81.1 (N-CH_2_-CO-O*C*(CH_3_)_3_), 71.0 (-N-CH_2_-*CH*-CO), 58.7 (N-*CH*_2_-CH-CO), 58.1 (N-*CH*_2_-CO-OC(CH_3_)_3_), 53.8, 52.9, 52.8.

### (HPA-DO3A)_2_ (H_2_L2)

Compound **2** (28 mg, 0.023 mmol) was dissolved in a 1:1 solution of DCM:TFA and stirred overnight at room temperature. After evaporation under reduced pressure, ligand **H**_2_**L2** was obtained in 95% yield (18 mg, 0.021 mmol). MS (ESI^+^): *m/z*: 447.8 [(M+2H)/2]^+^;^1^H NMR (500 MHz, CD_3_CN, 25°C): δ = 4.62 (dd, 2H; N-CH_2_-C*H*-CO), 4.12 (m, 4H; N-C*H*_2_-CH-CO), 3.61 (m, 12H; N-C*H*_2_-COOH, 12H), 3.15-3.50 (m, 32 H; cyclen,) ppm. ^13^C{^1^H}NMR (125MHz, CD_3_CN, 25°C): δ = 173.2 (N-CH_2_-CH-*CO*), 173.2 (N-CH_2_-*CO-*OH), 66.4 (N-CH_2_-*CH*-CO), 55.4 (N-*CH*_2_-CH-CO-), 54.4 (N-*CH2*-COOH), 53.2, 51.0, 50.3, 48.7, (cyclen), 38.6 (CO-NH-*CH*_2_*)* ppm.

### Ln(III) complexes

Ln(NO_3_)_3_ (Ln = Gd and Eu; 0.5 M aqueous solutions) was added to a solution of the ligand in water (2 mL). The pH was gradually adjusted to 7 and the solution stirred at room temperature overnight. The pH was then increased to 10 to precipitate excess Ln^III^ as hydroxide. The solution was centrifuged (4,000 rpm, 3 min, r.t.) and the supernatant filtered through a 0.2 μm filter. The pH was re-adjusted to 7 and the solvent removed in vacuo. Analytical HPLC: Pump A: H_2_O; Pump B = MeOH; flow = 1 mL/min; 0–3 min = 100% A; 3–18 min = 100% B. Gd_2_**L2**: t_r_ = 9.35 min. MS (ESI^+^): *m/z*: 600.98 [(M+2H)/2]^+^; Eu_2_**L2**: t_r_ = 9.01 min. MS (ESI^+^): *m/z*: 596.68 [(M+2H)/2]^+^. The concentration of Ln-complexes was assessed by using the Evan's method.

### Chemical exchange saturation transfer (CEST) experiment

Z-spectra of Eu_2_**L2** water solutions at different pH in a range between 4.0 and 9.1 were acquired at 21°C, 7T on a Bruker Avance 300 spectrometer equipped with a microimaging probe. A frequency offset range of ± 100 ppm was investigated. A typical RARE (Rapid Acquisition with Refocused Echoes) spin-echo sequence with TE 3 ms, TR 5 s and RARE factor 16 was used. An isotropic 64 × 64 acquisition matrix with a FOV of 12 mm and a slice thickness of 1 mm was used. The whole sequence was preceded by a saturation scheme consisting of a continuous rectangular wave pulse 2 s long with a radiofrequency B_1_ field of 12 μT. The Z-spectra were interpolated by smoothing splines to identify the zero-offset on a pixel-by-pixel basis of the bulk water and, then, to assess the correct ST% value over the entire range of frequency offsets investigated. Custom-made software, compiled in the Matlab platform (Mathworks Inc., Natick, MA), was used (Stancanello et al., 2008; Terreno et al., 2009). The extent of CEST effect was calculated as follows:

(1) $$\text{ST}\%=\left(1-\frac{{\text{M}}_{\text{s}}}{{\text{M}}_{0}}\right)\times 100$$

where MS is the intensity of the bulk water NMR signal after the irradiation on resonance (Δω) of the mobile proton pool and M0 is the intensity of the bulk water NMR signal after the irradiation at the opposite frequency (−Δω).

### Relaxometric measurements

The water proton longitudinal relaxation rates as a function of the magnetic field strength were measured in non-deuterated aqueous solutions on a Fast Field-Cycling Stelar SmarTracer relaxometer (Stelar s.r.l., Mede (PV), Italy) over a continuum of magnetic field strengths from 0.00024 to 0.25 T (corresponding to 0.01–10 MHz proton Larmor frequencies). The relaxometer operates under computer control with an absolute uncertainty in 1/*T*_1_ of ±1%. Additional longitudinal relaxation data in the range 20–70 MHz were obtained on a Stelar Relaxometer connected to a Bruker WP80 NMR electromagnet adapted to variable-field measurements. The exact concentration of Gd(III) was determined by measurement of bulk magnetic susceptibility shifts of a *t*BuOH signal or by inductively coupled plasma mass spectrometry (ICP-MS, Element-2, Thermo-Finnigan, Rodano (MI), Italy). Sample digestion was performed with concentrated HNO_3_ (70%, 2 mL) under microwave heating at 160°C for 20 min (Milestone MicroSYNTH Microwave lab station equipped with an optical fiber temperature control and HPR-1000/6 M six position high pressure reactor, Bergamo, Italy). The ^1^H *T*_1_ relaxation times were acquired by the standard inversion recovery method with typical 90° pulse width of 3.5 μs, 16 experiments of 4 scans. The temperature was controlled with a Stelar VTC-91 airflow heater equipped with a calibrated copper-constantan thermocouple (uncertainty of ±0.1°C).

Variable-temperature ^17^O NMR measurements were recorded on a Bruker Avance III spectrometer (11.7 T) equipped with a 5 mm probe and standard temperature control unit. Aqueous solutions of the complexes containing 2.0% of the ^17^O isotope (Cambridge Isotope) were used. The observed transverse relaxation rates were calculated from the signal width at half-height.

### MR-phantom imaging

MR images of capillaries filled with 1.5 mM water solutions of Gd_2_**L1**, Gd_2_**L2**, or ProHance were acquired at 21°C, 7 T on a Bruker Avance 300 spectrometer equipped with a microimaging probe. T_2W_ images were acquired by using a standard RARE (Rapid Acquisition with Refocused Echoes) sequence with the following parameters: TR = 5,000 ms, TE = 5.5 ms, FOV = 1 × 1 cm, slice thickness = 1 mm, RARE factor = 32, matrix size 128 × 128). T_1W_ images were acquired by using a standard MSME (multi-slice multi-echo) sequence with the following parameters: TR = 50 ms, TE = 3.3 ms, FOV = 1 × 1cm, slice thickness = 1 mm, matrix size 128 × 128). T_1_ values were measured by using a Saturation Recovery Spin Echo sequence with the following parameters: TE = 3.8 ms, 16 variable TR ranging from 50 to 5,000 ms, FOV = 1 × 1 cm, slice thickness = 1 mm).

### Theoretical modeling

Theoretical calculations were performed with Gaussian16 program (Frisch et al., 2016) at the density functional theory (DFT) level with the hybrid functional B3LYP (Becke, 1993), comprising a part of the exact exchange along with Becke's exchange and Lee-Yang-Parr correlation functionals. To limit the computational burden, the following effective core potentials were used for all the heavy atoms, along with the corresponding valence basis sets: LANL2DZ (Hay and Wadt, 1985a,b; Wadt and Hay, 1985) for C, N, O and MWB53 (Dolg et al., 1989) for Gd; long range solvent effects were computed for some systems through the polarizable continuum model (PCM) (Cossi et al., 2003); dispersion energies were included in all the calculations with the atom-atom semiempirical method and parameters proposed by Grimme (Grimme et al., 2010). When computing the complexation energies, Boys' counterpoise correction was applied to compensate the basis set superposition error (BSSE).

## Results and discussion

### Ligand design and synthesis

The ligand **L1** simply consists of two DOTA-monopropionamide units linked by an ethylene chain, therefore we expect that the relaxometric properties of the Gd complex would be consistent with a system with doubled molecular weight and similar electronic and water exchange parameters with respect to the monomeric GdDOTAMAP complex (Tei et al., 2009a). On the other hand, in case of the ligand **H**_2_**L2**, there is a substantial change with respect to HPDO3A: a hydroxypropylamide group replaced the hydroxypropyl group in order to generate electron withdrawing effect on the coordinated hydroxyl group and therefore stronger coordinating ability. Moreover, the hydrophilic nature of the amide group is expected to contribute to the overall relaxivity of the system through formation of hydrogen bonds to second sphere water molecules. The two monomeric hydroxypropylamideDO3A chelators were linked again by a short ethylene moiety.

**Scheme 2:**
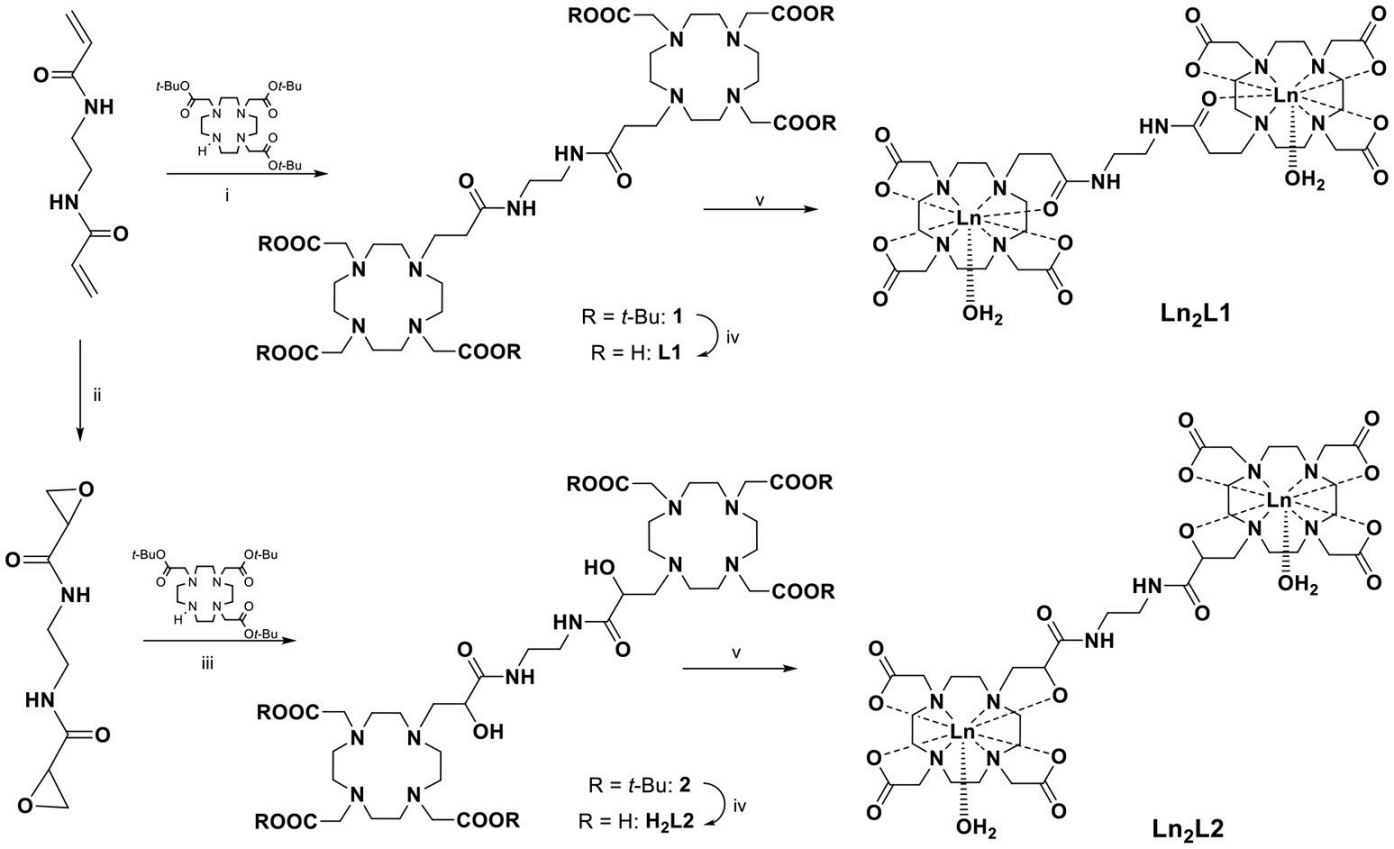
Synthesis of the ligands (DOTAMAP)_2_ (**L1**) and (HPADO3A)_2_ (**H**_2_**L2**) and their Ln(III) complexes (Ln = Gd, Eu). (i) DIPEA, DMF, 80°C, overnight; (ii) *m*-CPBA, CH_3_CN, MW (200 W, 95°C), 45 min; (iii) CH_3_CN, K_2_CO_3_, rt, overnight; (iv) DCM:TFA 1:1, rt, overnight; (v) Ln(NO_3_)_3_, H_2_O, pH 7, overnight.

Both chelates were obtained from a bis-acrylamide, ethylene-bis-acrylamide, through a multistep synthesis (Scheme 2). The synthesis of (DOTAMAP)_2_ (**L1**) started from the Michael addition of the bis-acrylamide to two equivalents of DO3A(*t*Bu)_3_ in dimethylformamide (DMF) in the presence of diisopropylethylamine (DIPEA) to give the protected ditopic ligand (**1**), which was immediately subjected to deprotection by reaction with a 1:1 mixture of trifluoroacetic acid (TFA) and dichloromethane (DCM). Then, complexation with Gd(NO_3_)_3_ was carried out in water at pH 7 and at room temperature. The purification by semi-preparative HPLC-MS was carried out on the final complex to obtain Gd_2_**L1** in 22% overall yield. The protected and deprotected ditopic intermediates were not isolated due to the tendency to elimination of the pendant arm to form again the acrylamide. This tendency is highly reduced in the presence of the Gd-complex, although solutions of Gd_2_**L1** left in water at pH 7 for more than one month revealed the presence of a small amount of elimination products. A similar approach was followed by Meade and co-workers (Rotz et al., 2015) for the synthesis of a *N*-propargylpropionamide derivative used for the preparation of Gd-labeled gold nanoparticles.

On the other hand, to obtain (HPA-DO3A)_2_ (**H**_2_**L2**), the bis-acrylamide was subjected to bis-epoxidation with meta-chloroperbenzoic acid (*m*-CPBA) in acetonitrile (ACN), using microwave heating (70 W, 90°C). After HPLC-MS purification the bis-epoxide was reacted with DO3A(*t*Bu)_3_ in ACN in the presence of K_2_CO_3_ to give the protected HPA-DO3A dimer (**2**) which was purified by column chromatography and finally deprotected with a 1:1 mixture of TFA and DCM. Intermediates and final ligand were characterized by ESI mass spectrometry and ^1^H and ^13^C NMR spectroscopy (Figures S7–S14). The Gd^III^ complex Gd_2_**L2** was prepared at room temperature by adding Gd(NO_3_)_3_ to a solution of **H**_2_**L2** while maintaining the pH at 6.5 with diluted NaOH. The excess of free metal ions in the solution was precipitated by the addition of NaOH up to pH 9 and the Gd-complex was isolated through a successive centrifugation, filtration, and lyophilization.

### Chemical exchange saturation transfer (CEST) measurements on Eu_2_L2

Lanthanide complexes of HPDO3A have been largely used as ParaCEST agents for cell labeling or extracellular/extravascular pH assessment in tumor models because the hydroxyl proton are close enough to the paramagnetic center to be able to generate CEST contrast (Nicholls et al., 2015; Pumphrey et al., 2016; Ferrauto et al., 2017). Moreover, as the exchange rate of the OH proton is pH dependent, these chelates have been exploited as pH sensitive ParaCEST agents (Delli Castelli et al., 2014). Since the p*K*_a_ of these hydroxyl protons can be modulated by inserting electron withdrawing or donating groups in close proximity, the CEST contrast may be used to determine the deprotonation of the hydroxyl group. In the binuclear Ln_2_**L2** complexes, the presence of an electron withdrawing amide group close to the hydroxyl group makes the OH proton very acid. Hence, it is reasonable to argue that this group is deprotonated at physiological pH values.

**Figure 1:**
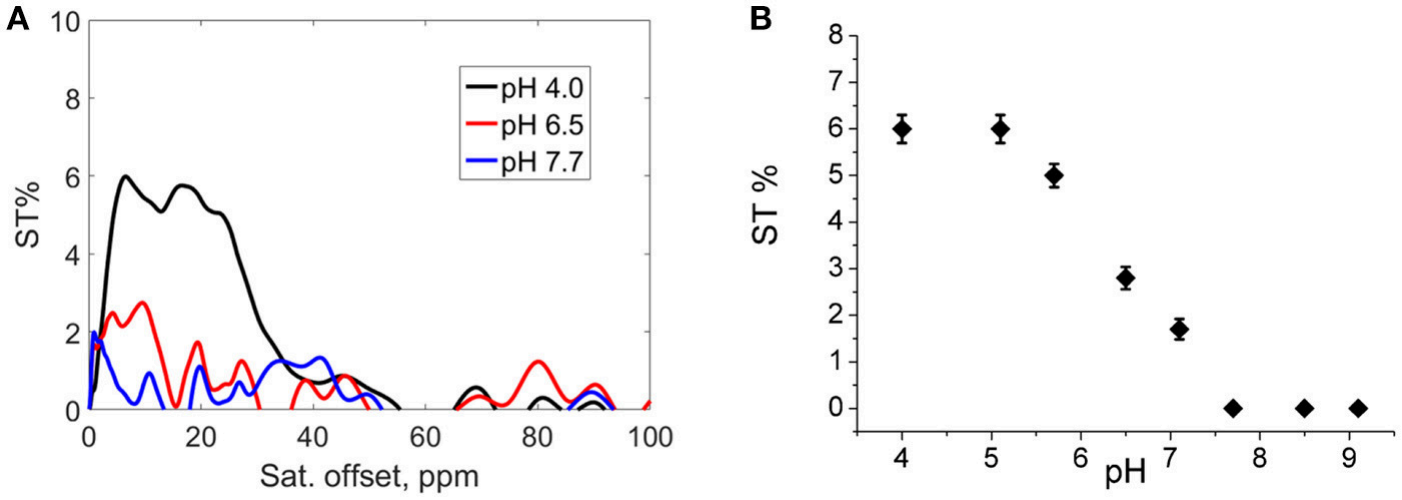
**(A)** ST-spectra of Eu_2_**L2** water solution at pH = 4.0 (black line), pH = 6.5 (red line) and pH = 7.7 (blue line); **(B)** ST% vs. pH. (Experimental set-up: B_1_ = 12 μT, [Eu_2_**L2**] = 5 mM, T = 21°C).

In order to obtain indirect insights into the exchangeable protons of the hydroxyl moieties, Z-and ST-spectra of Eu_2_**L2** water solutions at different pH in a range between 4.0 and 9.1 were acquired. In CEST experiment, the resonance of exchangeable protons can be specifically saturated by a proper *rf* pulse. Saturated spins are transferred to bulk water through chemical exchange. In such a way, an indirect saturation of *bulk* water signal occurs, that can be observed in a ^1^H-MR image. Z-spectra reports about bulk water signal as a function of the *rf* offset (Supplementary information, Figure S1). From Z-spectra, the symmetrical analysis allows obtaining ST-spectra, in which saturation transfer is plotted against the *rf* offset. Representative ST-spectra of Eu_2_**L2** water solution at pH 4.0, 6.5 and 7.7 are reported in Figure 1A. The CEST effect is clearly visible at acid pH, barely visible at pH = 6.5 and completely absent at pH > 7.5. The ST% effect vs. pH is reported in Figure 1B showing the disappearance of CEST effect at neutral pH. Thus, this result is in line with the hypothesis of hydroxyl deprotonation at these pH values.

### Relaxometric measurements

The published value of *r*_1_ for GdDOTAMAP-En (Tei et al., 2009a) and GdHPDO3A (Delli Castelli et al., 2013), at 0.47 T and 298 K, are 4.9 and 4.6 mM^−1^ s^−1^, respectively. These are typical values of the clinical MRI contrast agents, i.e., monohydrated low-molecular weight Gd^III^ chelates that tumble rapidly in solution and whose effectiveness as relaxation agents at high fields (≥ 0.47 T) is defined primarily by their rotational dynamics and thus by their molecular mass. In fact, the different *r*_1_ values are associated with different values of τ_R_, namely 79 and 65 ps for GdDOTAMAP-En and GdHPDO3A, respectively. The ionic (per Gd) *r*_1_ values for the corresponding dimeric complexes, measured under identical experimental conditions, are 8.7 and 9.5 mM^−1^ s^−1^ for Gd_2_**L1** and Gd_2_**L2**, respectively. These values remain almost constant in the pH range 2–10 (Figure S2). These values correspond to relaxivity increases of + 77% and + 106% relative to the *r*_1_ values of the corresponding mononuclear GdDOTAMAP-En and GdHPDO3A complexes. The relaxivity gain can be easily attributed to a longer value of the rotational correlation time associated with the increased molecular size, combined with the preservation of the same hydration state (*q* = 1) for each metal ion (Aime et al., 1992). From this information, we deduce that the binuclear complexes are remarkably rigid and compact, with a low degree of rotational flexibility about the linker. However, considering that only the inner-sphere contribution to relaxivity depends on τ_R_, while the outer-sphere contribution remains almost unchanged, the particularly strong *r*_1_ enhancement measured for Gd_2_**L2** might suggest the presence of an additional contribution.

**Figure 2:**
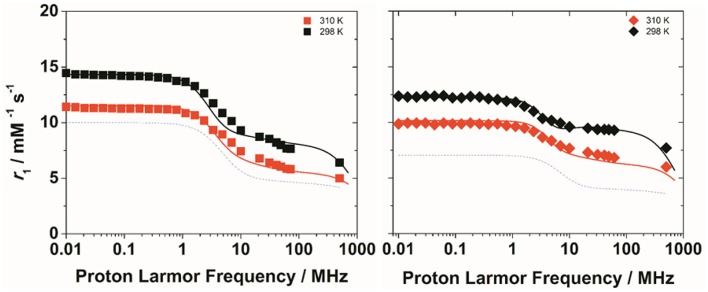
^1^H NMRD profiles of Gd_2_**L1** (left) and Gd_2_**L2** (right) recorded at 298 K (black symbols) and 310 K (red symbols) and pH 7. The solid lines represent the best fitting results of the experimental data points with the parameters in Table 1, Model 1. The dotted lines correspond to the NMRD profile at 298 K of GdDOTAMAP-En (left) and GdHPDO3A (right).

To get more insight into the physico-chemical characteristics of these novel ditopic complexes, a detailed ^1^H and ^17^O NMR relaxometric study was carried out. The magnetic field dependence of *r*_1_, the so-called nuclear magnetic relaxation dispersion (NMRD) profiles, were measured at 25 and 37°C in the proton Larmor frequency range 0.01–500 MHz, corresponding to magnetic field strengths varying between 2.34 × 10^−4^ T and 11.7 T (Figure 2). The shape of the NMRD profiles and their temperature dependence (*r*_1_ decreases with increasing temperature, Figures S3, S4) reproduce the general behavior of small Gd^III^ complexes, characterized a plateau at low fields, a dispersion around 4–6 MHz and another region at high fields (> 20 MHz) where *r*_1_ is almost constant or changes very little. The lower values of the relaxivity at 37°C, over the entire range of proton Larmor frequencies investigated, indicate that *r*_1_ is not limited by the water exchange rate (*fast exchange* regime) but rather by the rotational motion, as for the related monomeric complexes. A least-square fit of the profiles was carried out in terms of the established theory of paramagnetic relaxation expressed by the Solomon-Bloembergen-Morgan (Bloembergen and Morgan, 1961) and Freed's (Freed, 1978) equations for the *inner*- (IS) and *outer sphere* (OS) proton relaxation mechanisms, respectively. Because of the large number of parameters involved in the fitting procedure, some of them are usually fixed to known or reasonable values. The hydration number *q* was fixed to 1; the distance between Gd^3+^ and the protons of the bound water molecule, *r*, was fixed to 3.0 Å; the distance of closest approach, *a*, of the *outer sphere* water molecules to Gd^3+^ was set to 4.0 Å and for the relative diffusion coefficient *D* standard values of 2.24 and 3.1 × 10^−5^ cm^2^ s^−1^ (298 and 310 K) were used. The fit was performed using as adjustable parameters τ_R_ and the electronic relaxation parameters Δ^2^ (trace of the squared zero-field splitting, ZFS, tensor) and τ_V_ (correlation time for the modulation of the transient ZFS).

**Figure 3:**
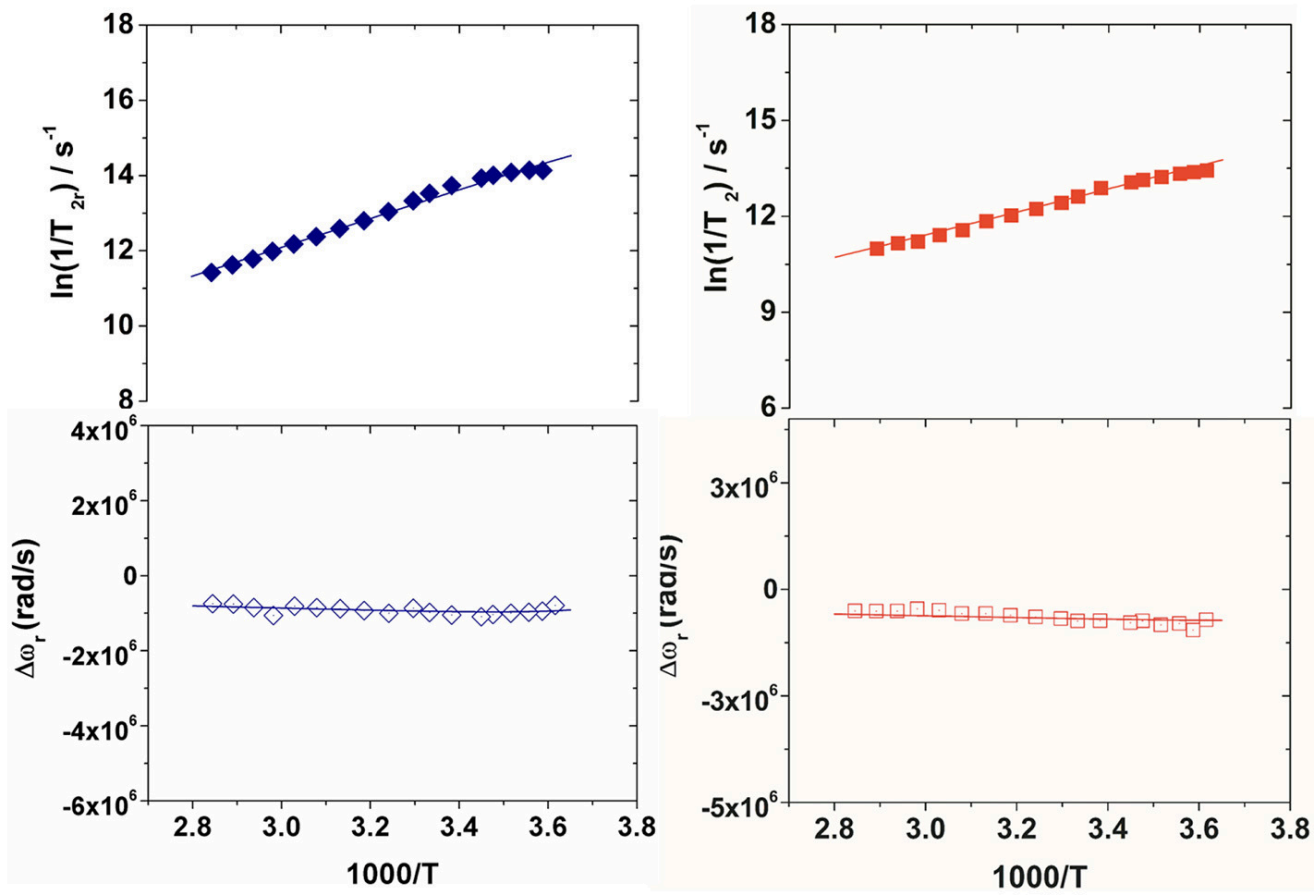
Reduced transverse ^17^O relaxation rates (top) and chemical shifts (bottom) measured at 11.74 T (pH 7) for Gd_2_**L1** (left) and Gd_2_**L2** (right). The solid lines correspond to the fits of the data as described in the text.

**Figure 4:**
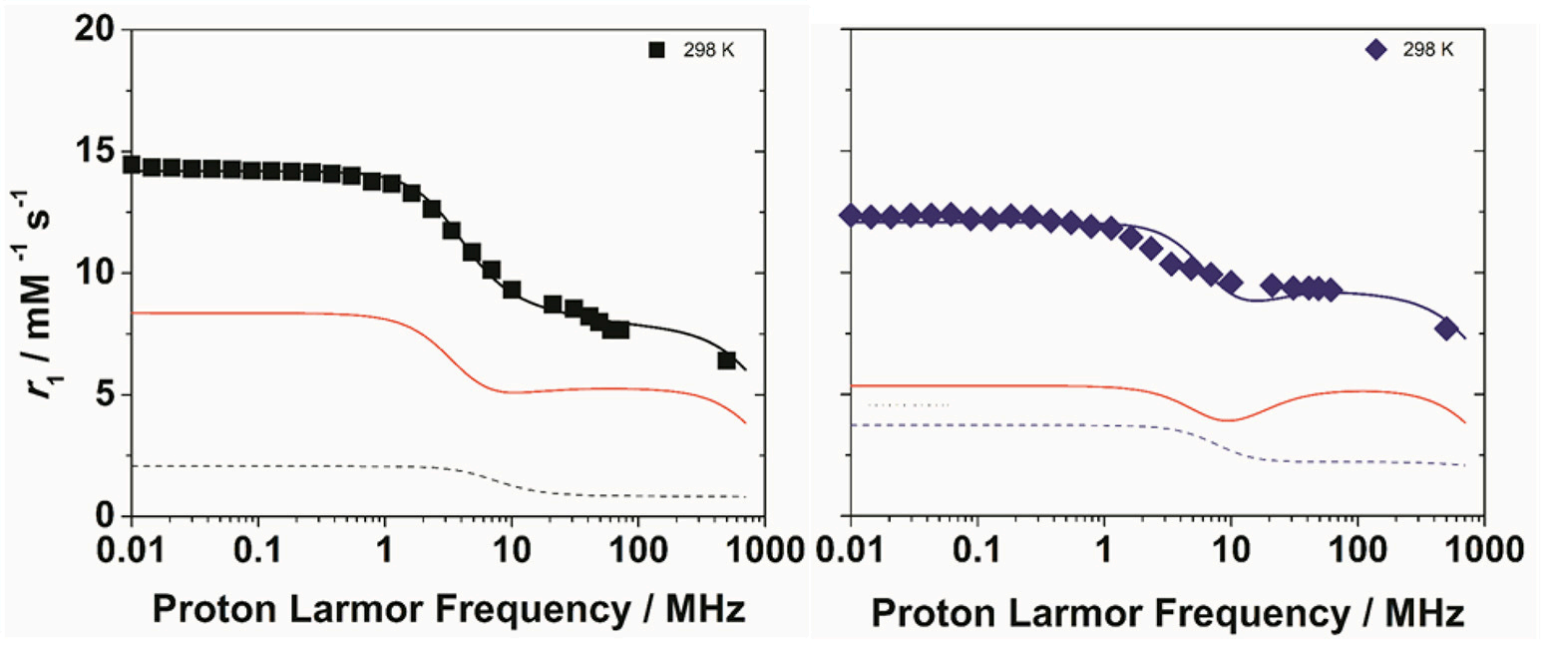
^1^H NMRD profiles of Gd_2_**L1** (left) and Gd_2_**L2** (right) recorded at 298 K and pH 7. The solid lines represent the best fitting results of the experimental data points with the parameters in Table 1, Model 2. The red and dotted lines correspond to the inner and second sphere contributions to the relaxivity, respectively.

The residence lifetime of coordinated water, τ_M_, does not affect the relaxivity of the systems under the regime of fast exchange. However, the accurate value of this parameter can be obtained through the measurement of the temperature dependence of the ^17^O NMR transverse relaxation rate, *R*_2_, and paramagnetic shift, Δω, of the solvent water. The data were measured at 11.7 T on 20 and 16 mM solutions of Gd_2_**L1** and Gd_2_**L2**, respectively, at neutral pH. The experimental data are often reported as reduced transverse relaxation rates, *R*_2r_, defined as 1/*T*_2r_ = *R*_2r_ = *R*_2p_/*p*_M_, where *p*_M_ is the molar fraction of inner-sphere water molecules. The reduced transverse ^17^O-relaxation rates and chemical shifts (Δω_r_) measured for Gd**L1** and Gd**L2** are reported in Figures 3, 4. In both cases 1/*T*_2r_ increases with decreasing temperature over the temperature range studied (275-352 K), indicating high rate of exchange for the bound water molecule. The data were analyzed in terms of the Swift-Connick theory for ^17^O relaxation (Swift and Connick, 1962) using as fitting parameters Δ^2^, τ_V_, the τ_M_ value at 298 K, its enthalpy of activation ΔH_M_, the scalar Gd-^17^O_w_ coupling constant *A*/h. Moreover, the temperature dependence of τ_V_ and τ_R_ has been considered through their activation energies *E*_V_, set to 1.0 *k*J mol^−1^, and *E*_R_, fixed to 18.0 *k*J mol^−1^. The best-fit parameters are listed in Table 1 and compared with those previously reported for the related mononuclear complexes.

**Table 1:** Best-fit parameters obtained from the analysis of the 1/*T*_1_^1^H NMRD profiles (298 and 310 K) and ^17^O NMR data for GdHPDO3A,^a^ GdDOTAMAP-En,^b^ Gd_2_**L1** and Gd_2_**L2**^c^.

**Parameter**	**GdHPDO3A**		**GdDOTAMAP-En**	**Gd**_**2**_**L1**		**Gd**_**2**_**L2**	
	**SAP**	**TSAP**		**Model 1**	**Model 2**	**Model 1**	**Model 2**
				**(IS+OS)**	**(IS+OS+SS)**	**(IS+OS)**	**(IS+OS+SS)**
^298^*r*_1_ (mM^−1^ s^−1^) 20 MHz	4.6		4.9	8.7		9.5	
^310^*r*_1_ (mM^−1^ s^−1^) 20 MHz	3.6		3.9	6.8		7.3	
^298^τ_M_ (ns)	640	8.9	12	10.0 ± 1.2		5.0 ± 0.4	
^298^τ_R_ (ps)	65		79	169 ± 4	140 ± 2	205 ± 3	140^c^
Δ^2^ (10^19^ s^−2^)	9.9	1.5	3.9	1.3 ± 0.1	1.8 ± 0.1	2.0 ± 0.1	6.2 ± 0.3
^298^τ_v_ (ps)	8	30	15	40 ± 2	36 ± 2	40 ± 2	22 ± 1
Δ*H_*M*_* (*k*J mol^−1^)	53^d^	15^d^	29.7	30.6 ± 1.1		29.5 ± 1.1	
*A/h* (10^6^ rad s^−1^)	−3.5		−3.2	−3.5 ± 0.1		−3.4 ± 0.1	
*q*_SS_	–		–	–	2	–	4
^298^τ_R(SS)_ (ps)	–		–	–	45 ± 2	–	60 ± 4

a From Delli Castelli et al. (2013);

b from Tei et al. (2009a);

c The parameters fixed in the fitting procedure are: q = 1, r_GdO_ = 2.5 Å, r_GdH_ = 3.0 Å, a_GdH_ = 4.0 Å, ^298^D_GdH_ = 2.24 × 10^−5^ cm^2^ s^−1^, E_R_ = 18 kJ mol^−1^, Ev = 1 kJ mol^−1^, r_GdH(SS)_ = 3.8 Å;

d *Activation energy, E*.

The *k*_ex_ value obtained for Gd_2_**L1** (1.0 × 10^8^ s^−1^; Table 1) is quite similar to that of the parent GdDOTAMAP-En complex, indicating that the formation of the ditopic complex did not significantly alter the coordination geometry around the metal ion. In the case of Gd_2_**L2**, the residence lifetime of the bound water molecule is quite short (5.0 ns) thus suggesting that, unlike the case of the monomeric GdHPDO3A complex, the formation of the dimer involves the occurrence of a predominant population of fast-exchanging TSAP isomer.

**Figure 5:**
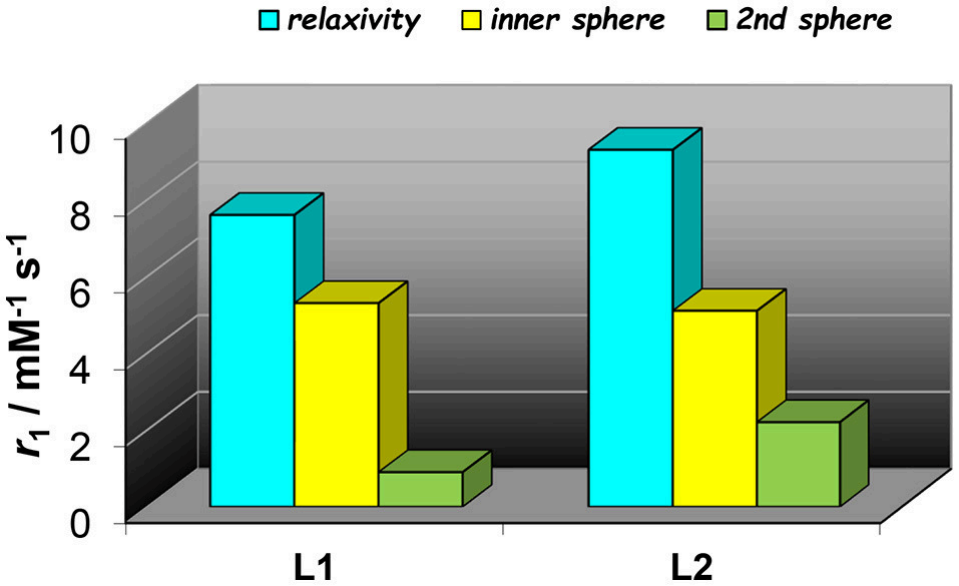
Comparison of proton relaxivities of Gd_2_**L1** and Gd_2_**L2** (298 K, 60 MHz) showing the inner- and second sphere contributions as analyzed with Model 2.

The experimental NMRD curves were first analyzed with a model (Model 1) that takes into account the presence of only IS and OS contributions to relaxivity. The profiles are rather well reproduced with the set of parameters listed in Table 1, which clearly show that the *r*_1_ values of the dimeric complexes and their frequency dependence may be attributed predominantly to the slowdown of the rotational motion and then to the longer τ_R_ values. For Gd_2_**L1** the increase in the τ_R_ value with respect to the value reported for GdDOTAMAP-En is 114%. Therefore, the binuclear complex presents a remarkable stereochemical rigidity and the greater molecular mass, compared to the monomer, is entirely translated into a corresponding lengthening of τ_R_. In the case of Gd_2_**L2** the value of τ_R_ is 215% longer than the value found for GdHPDO3A. Since the increase in the molecular mass is only of 114% is clear that the *r*_1_ enhancement must be favored by an additional contribution. This might be identified in a sizeable second-sphere (SS) contribution, which corresponds to the presence of water molecules hydrating the complex at a distance from Gd^3+^ sufficiently short (ca. < 4 Å) and with a residence time sufficiently long to be affected by the rotation (Botta, 2000). We analyzed the NMRD profiles considering also this possible contribution, expressed in terms of two additional parameters: the number *q*_SS_ of second sphere water molecules and their rotational correlation time, τ_R(SS)_ (Model 2 in Table 1). The average distance from the paramagnetic center has been arbitrarily fixed at 3.5 Å, an intermediate value between those of water molecules in the inner (3.0 Å) and outer (4.0 Å) solvation shell (Figure 4). In the case of Gd_2_**L1** the best results are obtained by taking into account the contribution of two water molecules belonging to the SS and characterized by a rotational correlation time τ_R(SS)_ of 45 ps. This involves consequently a decrease in the global τ_R_ passing from 169 to 140 ps, a value that best fits the molecular size of the complex. The same value of τ_R_ was used in the analysis of the NMRD profile of Gd_2_**L2**, obtaining the best result with the additional contribution of four SS water molecules with a τ_R(SS)_ of 60 ps. As it is clearly apparent from the plot of Figure 5, the SS contribution is rather small in the case of Gd_2_**L1**, about 12% at 1.5 T and 310K, while it turns out to be very significant for Gd_2_**L2** as it can be attributed to it over 20% of the overall relaxivity measured. The greater weight of the SS contribution to Gd_2_**L2** is probably due to the presence of negative charges in the complex due to the deprotonation of the alcohol groups, in accordance with the CEST data.

### MR phantom images

**Figure 6:**
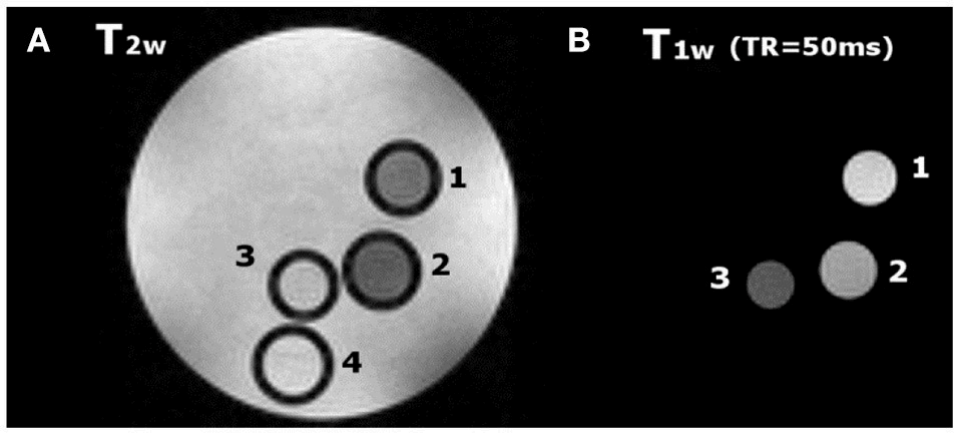
**(A)** T_2w_ image (TR = 5 s, TE = 5.5 ms) and **(B)** T_1w_ image (TR = 50 ms, TE = 3.3 ms) of a phantom containing glass capillaries filled with: (1) (Gd_2_**L2** 1.5 mM, 2); (Gd_2_**L1** 1.5 mM, 3); GdHPDO3A 1.5 mM and, (4) water.

MR images of phantom containing glass capillaries filled with 1.5 mM water solutions of Gd_2_**L1**, Gd_2_**L2**, or GdHPDO3A were imaged at 7 T. T_2w_ and T_1w_ representative images are reported in Figures 6A,B, respectively. The Signal Intensity in T_1w_ image is higher in the two capillaries filled with dinuclear Gd-complexes (capillary 1 and 2 in Figure 6) with respect to that one filled with GdHPDO3A (capillary 3 in Figure 6). Furthermore, as expected, contrast enhancement provided by Gd_2_**L2** is visibly higher than that one provided by Gd_2_**L1**.

### Molecular modeling on Gd_2_L2

**Figure 7:**
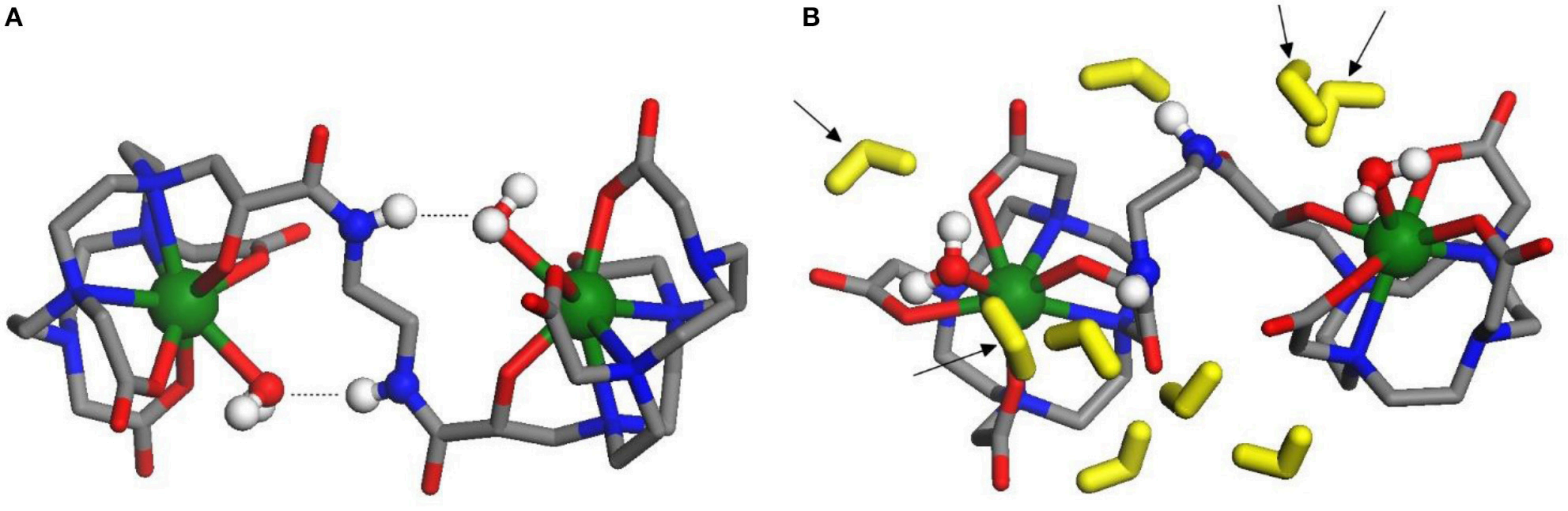
(**A**, left) Optimized structure of dinuclear bis-aqua [Gd_2_**L2**(H_2_O)_2_]^2−^ in vacuo: the amide-coordinated water H-bonds are indicated by dotted lines. Green, Gd; blue, N; gray, C; red, O. in vacuum; (**B**, right) Optimized structure of dinuclear bis-aqua [Gd_2_**L2**(H_2_O)_2_]^2−^ in water: the explicit water molecules in the second shell are colored in yellow; arrows indicate the 4 molecules of the second shell closest to Gd ions. Green, Gd; blue, N; gray, C; red, O. in water.

The geometry of isolated Gd_2_**L2** dimer was optimized at the DFT level, including a water molecule coordinated to each Gd^3+^ ion; to simulate the conditions at physiological pH, the carboxylic and hydroxyl groups on both HPADO3A chelators were deprotonated, generating a double negative charge on the dimer. Then, the solvent (water) effects were included, re-optimizing the geometry with 9 water molecules in the second solvation shell and using the PCM model to account for the long range electrostatic interactions with the rest of the solvent. The optimized structures of the isolated and solvated dimer are depicted in Figures 7A,B, respectively. In both cases, one water molecule is coordinated to each metal ion: the complexation energies (obtained by removing one of the coordinated waters and recomputing the energy of the resulting dimer and of the water molecule alone) resulted −33.6 *k*J/mol in vacuo and −22.0 *k*J/mol in the solvent. Not surprisingly, solvent effects weaken the coordination bond since the separated fragments, especially the charged Gd^III^ complex, can be “surrounded” by the polarized continuum better than the initial structure, so their solvation energy is higher.

In the isolated dimer (Figure 7A), due to the H-bonds between amide groups and coordinated waters, the dihedral angle of the N-C-C-N branch connecting the two GdHPADO3A moieties is close to 180°, so that the Gd-H_2_O bond directions are almost opposite to each other. This arrangement changes when the solvent is added, since the amide groups prefer to bind to water molecules in the second solvation shell, which are more free to move improving the H-bond stability: as a consequence, the dihedral angle is optimized around 104° and the Gd-H_2_O bonds are much more parallel than in the former case. The distance of the coordinated water hydrogen atoms from Gd is 2.8/2.9 Å both in vacuo and in solution. In the latter structure, two water molecules of the second solvation shell are quite close to each GdHPDO3A moiety (indicated by arrows in Figure 7B): the distance of their hydrogens from Gd is 3.2–4.8 Å, a range in fair agreement with the NMR relaxometric results above described. It is also interesting to compute the energy profile for the rotation of the GdHPDO3A ends around the N-C-C-N branch, since this parameter has been shown to be critical in assessing the relaxation efficacy. Thus, we have performed a rigid scan of the dinuclear Gd_2_**L2** complex with respect to this dihedral angle in vacuo (Figures S5) and in water (Figure S6): in the latter case, each second shell water molecule was attributed to the closer GdHPDO3A moiety and moved along with it during the scan. The energy curves along this rotation are quite different in vacuo and in the presence of solvent molecules. As discussed above, the isolated dimer is stabilized when the Gd-cages are oriented in opposite directions, and a barrier of around 40 kJ mol^−1^ is required to break the intramolecular H-bonds and rotate the two units. On the other hand, more than one energy minimum is found for the solvated dimer, reflecting the larger number of intermolecular interactions available with the water molecules. The computed rotational barrier is higher, around 120 kJ mol^−1^, although this result is likely overestimated for the presence of a limited number of solvent molecules, which do not describe the solvation shell for all the conformations with the same accuracy. In any case, we can safely conclude that a high rotational barrier is also expected in aqueous solution.

## Conclusions

Dinuclear Gd^III^ and Eu^III^ complexes based on two well-established *q* = 1 monomeric chelates derived from the macrocyclic systems HPDO3A and DOTAMAP were successfully synthesized via a multi-step procedure. The macrocyclic structure of the complexes guarantees the excellent thermodynamic stability and kinetic inertness of the parent complexes. While the Ln_2_**L1** complexes are electrically neutral, with the propionamide moiety coordinating the Ln-center, from CEST measurements on the Eu_2_**L2** complex we could conclude that the hydroxyl groups of the ligand **H**_2_**L2** deprotonates around physiological pH and coordinate tightly the metal center; hence, the Ln_2_**L2** complexes are dianionic. This hypothesis finds further support on the ^1^H and ^17^O relaxometric data, which showed a fast water exchange rate in Gd_2_**L2**, much faster than that found for the neutral GdHPDO3A, and a sizeable (ca. 20%) contribution of second sphere water molecules present in the surroundings of the GdHPADO3A cage. A model in which four SS water molecules at a relatively short distance from the Gd-center and with τ_R(SS)_ of 60 ps was considered to account for the large relaxivity enhancement found for the dinuclear Gd_2_**L2** with respect to the mononuclear GdHPDO3A (*r*_1_ = 9.5 vs. 4.6 mM^−1^s^−1^, at 20 MHz and 298 K). This additional contribution was necessary because the relaxivity gain exceeds the increase in the molecular correlation time associated with the corresponding increase in molecular size. In the case of Gd_2_**L1**, both models of analysis provide reasonable results, thus indicating the occurrence of a much lower SS contribution. The relaxometric results also confirm a high rigidity of both dinuclear complexes with a hindered or slow rotation through the linker connecting the two cages. These conclusions are further supported by the molecular modeling at the DFT level on Gd_2_**L2**, which identifies a group of water molecules in well-defined positions around the metal center and at a distance quite comparable with that estimated from relaxometric data. In addition, the calculations highlight the occurrence of a high energy barrier for the rotation through the C-C bond of the ethylene linker.

These experimental results confirm that simple systems like dinuclear Gd-complexes afford optimal results in terms of relaxivity enhancement at high field strengths, as shown by the phantom MR-images at 7 T, provided they are compact, stereochemically rigid, characterized by predominantly isotropic rotational motion. An additional relevant contribution arises from the presence of a well-defined second hydration sphere. Gd_2_**L2** features all these characteristics that provide an efficacy as relaxation agent over 110% higher (per Gd) than that of the clinically used MRI probes.

## Ethics statement

As the study presented in the manuscript does not involve human or animal subjects, an ethics approval was not required as per institutional and national guidelines.

## Author contributions

LL: performed chemical synthesis and relaxometric measurements; GF: performed the CEST experiments and the MR phantom Images. MC: performed the DFT study; LT, MB, and MC: performed interpretation of data and critically reviewed the manuscript; LT and MB: wrote the manuscript. All authors approved the final version of the manuscript.

### Conflict of interest statement

The authors declare that the research was conducted in the absence of any commercial or financial relationships that could be construed as a potential conflict of interest. The reviewer, EC, and handling Editor declared their shared affiliation.
