# Supplementary material for: Optimizing the Relaxivity of MRI Probes at High Magnetic Field Strengths With Binuclear GdIII Complexes
Source: Front Chem. 2018 May 15;6:158. doi: 10.3389/fchem.2018.00158 (PMC5962812; doi:10.3389/fchem.2018.00158)
Supplement: Supplementary file 1 [file Table_1.PDF]

# Supporting information

For

# Optimizing the relaxivity of MRI probes at high magnetic field strengths with binuclear $Gd^{III}$ complexes

Loredana Leone,[a] Giuseppe Ferrauto,[b] Maurizio Cossi,[a] Mauro Botta[a*] and Lorenzo Tei[a*]

[a] Dipartimento di Scienze e Innovazione Tecnologica (DiSIT), Università degli Studi del Piemonte Orientale "Amedeo Avogadro", Viale T. Michel 11, I-15121 Alessandria, Italy.

[b] Department of Molecular Biotechnology and Health Sciences, Molecular Imaging Centre, University of Torino, Via Nizza 52, 10126 Torino, Italy.

**Contents:**

1. Equations used for the analysis of $^{17}O$ NMR and NMRD data pag 2
2. Supplementary figures pag 5
3. $^{1}H$ and $^{13}C$ NMR spectra and LC-MS chromatograms pag 8
4. References pag 19

## 1. Equations used for the analysis of $^{17}$O NMR and NMRD data

### 1.1. $^{17}$O NMR spectroscopy:

From the measured $^{17}$O NMR transversal relaxation rates and angular frequencies of the paramagnetic solutions, $1/T_1$, $1/T_2$ and ω, and of the acidified water reference, $1/T_{1A}$, $1/T_{2A}$ and $\omega_A$, one can calculate the reduced relaxation rates, $1/T_{1r}$, $1/T_{2r}$ and reduced chemical shifts (Eq. (1) – (2)), where $1/T_{2m}$ is the relaxation rate of the bound water and $\Delta\omega_m$ is the chemical shift difference between bound and bulk water, $\tau_m$ is the mean residence time or the inverse of the water exchange rate $k_{ex}$ and $P_m$ is the mole fraction of the bound water.[i,ii]

$$\frac{1}{T_{2r}} = \frac{1}{P_m}\left[\frac{1}{T_2} - \frac{1}{T_{2A}}\right] = \frac{1}{\tau_m}\frac{T_{2m}^{-2} + \tau_m^{-1}T_{2m}^{-1} + \Delta\omega_m^2}{\left(\tau_m^{-1} + T_{2m}^{-1}\right)^2 + \Delta\omega_m^2} + \frac{1}{T_{2OS}} \tag{1}$$

$$\Delta\omega_r = \frac{1}{P_m}(\omega - \omega_A) = \frac{\Delta\omega_m}{(1+\tau_m T_{2m}^{-1})^2 + \tau_m^2\Delta\omega_m^2} + \Delta\omega_{os} \tag{2}$$

The outer sphere contributions to the $^{17}$O relaxation rates and chemical shifts have been considered negligible in the present study. $\Delta\omega_m$ is determined by the hyperfine or scalar coupling constant, $A/\eta$, according to Equation (3), where $B$ represents the magnetic field, S is the electron spin (S = 7/2 for high-spin Gd(II) complexes) and $g_L$ is the isotropic Landé $g$ factor.[iii]

$$\Delta\omega_m = \frac{g_L \mu_B S(S+1)B}{3k_B T}\frac{A}{\eta} \tag{3}$$

The exchange rate is supposed to assume the Eyring equation. In Eq. (4) $\Delta S^{\ddagger}$ and $\Delta H^{\ddagger}$ are the entropy and enthalpy of activation for the water exchange process, and $k_{ex}^{298}$ is the exchange rate at 298.15 K.

$$\frac{1}{\tau_m} = k_{ex} = \frac{k_B T}{h}\exp\left\{\frac{\Delta S^{\ddagger}}{R} - \frac{\Delta H^{\ddagger}}{RT}\right\} = \frac{k_{ex}^{298}T}{298.15}\exp\left\{\frac{\Delta H^{\ddagger}}{R}\left(\frac{1}{298.15} - \frac{1}{T}\right)\right\} \tag{4}$$

In the transverse relaxation the scalar contribution, $1/T_{2sc}$, is the most important, Eq. (5). $1/\tau_{s1}$ is the sum of the exchange rate constant and the electron spin relaxation rate.

$$\frac{1}{T_{2m}} \cong \frac{1}{T_{2SC}} = \frac{S(S+1)}{3}\left(\frac{A}{h}\right)^2 \tau_{S1} \tag{5}$$

$$\frac{1}{\tau_{S1}} = \frac{1}{\tau_m} + \frac{1}{T_{1e}} \tag{6}$$

## $^{1}$H NMRD

The measured longitudinal proton relaxation rate, $R_1^{obs}$ is the sum of a paramagnetic and a diamagnetic contribution as expressed in Eq. (7), where $r_{1p}$ is the proton relaxivity:

$$R_1^{obs} = R_1^d + R_1^p = R_1^d + r_{1p}[Gd(III)] \quad (7)$$

The relaxivity can be divided into an inner and an outer sphere term as follows:

$$r_1 = r_{1is} + r_{1os} \quad (8)$$

The inner sphere term is given in Eq. (9), where $q$ is the number of inner sphere water molecules.[iv]

$$r_{1is} = \frac{1}{1000} \times \frac{q}{55.55} \times \frac{1}{T_{1m}^H + \tau_m} \quad (9)$$

The longitudinal relaxation rate of inner sphere protons, $1/T_{1m}^{H}$ is expressed by Eq. (10):

$$\frac{1}{T_{1m}^H} = \frac{2}{15}\left(\frac{\mu_0}{4\pi}\right)^2 \frac{\gamma_I^2 g^2 \mu_B^2}{r_{GdH}^6} S(S+1)\left[\frac{3\tau_{d1}}{1+\omega_I^2\tau_{d1}^2} + \frac{7\tau_{d2}}{1+\omega_S^2\tau_{d2}^2}\right] \quad (10)$$

where $r_{GdH}$ is the effective distance between the electron charge and the $^{1}$H nucleus, $\omega_I$ is the proton resonance frequency and $\omega_S$ is the Larmor frequency of the Gd(III) electron spin.

$$\frac{1}{\tau_{di}} = \frac{1}{\tau_m} + \frac{1}{\tau_R} + \frac{1}{T_{ie}} \qquad i = 1\,,\,2 \quad (11)$$

The longitudinal and transverse electronic relaxation rates, $1/T_{1e}$ and $1/T_{2e}$ are expressed by Eqs. (12)-(14),[v] where $\tau_V$ is the electronic correlation time for the modulation of the zero-field-splitting interaction, $E_V$ the corresponding activation energy and $\Delta^2$ is the mean square zero-field-splitting energy. We assumed a simple exponential dependence of $\tau_V$ versus $1/T$ as written in Eq. (14).

$$\frac{1}{T_{1e}} = \frac{1}{25}\Delta^2\tau_V\,\{4S(S+1)-3\}\left(\frac{1}{1+\omega_S^2\tau_V^2} + \frac{4}{1+4\omega_S^2\tau_V^2}\right) \quad (12)$$

$$\frac{1}{T_{2e}} = \left(\left(0.02\times\left(4S^2+4S-3\right)\times\tau_V\times\Delta^2\times\left(\left(\frac{5}{1+\omega_S{}^2\tau_V{}^2}\right)\right)\right)+\left(\frac{2}{1+4\omega_S{}^2\tau_V{}^2}\right)+3\right) \quad (13)$$

$$\tau_v = \tau_v^{298}\exp\left\{\frac{E_v}{R}\left(\frac{1}{T} - \frac{1}{298.15}\right)\right\} \quad (14)$$

The outer-sphere contribution can be described by Eq. (15) where $N_A$ is the Avogadro constant, and $J_{os}$ is its associated spectral density function.[vi,vii]

$$r_{1os} = \frac{32 N_A \pi}{405}\left(\frac{\mu_0}{4\pi}\right)^2 \frac{\eta^2 \gamma_S^2 \gamma_I^2}{a_{GdH} D_{GdH}} S(S+1)\left[3J_{os}(\omega_I;T_{1e}) + 7J_{os}(\omega_I;T_{2e})\right] \qquad (15)$$

$$J^{OS}\left(\omega, T_{je}\right) = \mathrm{Re}\left[\frac{1+\frac{1}{4}\left(i\omega\tau_{GdH} + \frac{\tau_{GdH}}{T_{je}}\right)^{1/2}}{1+\left(i\omega\tau_{GdH} + \frac{\tau_{GdH}}{T_{je}}\right)^{1/2} + \frac{4}{9}\left(i\omega\tau_{GdH} + \frac{\tau_{GdH}}{T_{je}}\right) + \frac{1}{9}\left(i\omega\tau_{GdH} + \frac{\tau_{GdH}}{T_{je}}\right)^{3/2}}\right] \qquad (16)$$

where $j = 1,2$, $\tau_{GdH} = \frac{a_{GdH}{}^2}{D_{GdH}}$.

The diffusion coefficient for the diffusion of a water proton away from a Gd(III) complex, $D_{GdH}$, is assumed to obey an exponential law versus the inverse of the temperature, with an activation energy $E_{GdH}$, as given in Eq. (17). $D_{GdH}{}^{298}$ is the diffusion coefficient at 298.15 K.

$$D_{GdH} = D_{GdH}^{298} \exp\left\{\frac{E_{GdH}}{R}\left(\frac{1}{298.15} - \frac{1}{T}\right)\right\} \qquad (17)$$

## 2. Supplementary Figures

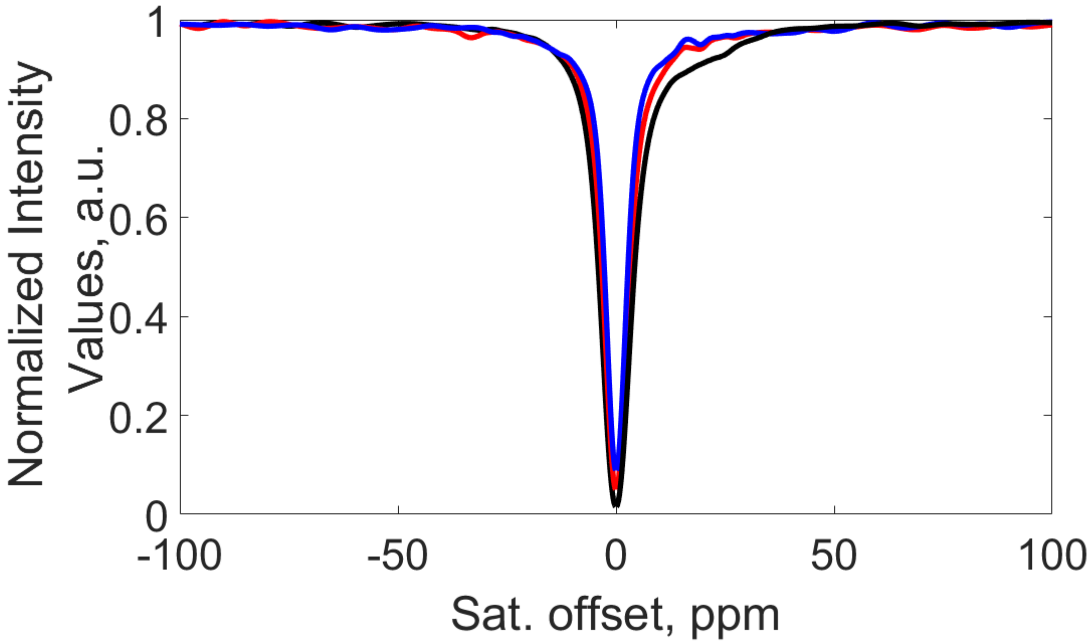


**Fugure S1.** Z-spectra of 5 mM solution of $Eu_2$**L2** at variable pH (from pH 4 to pH 9.1, $B_1$=12 μT).

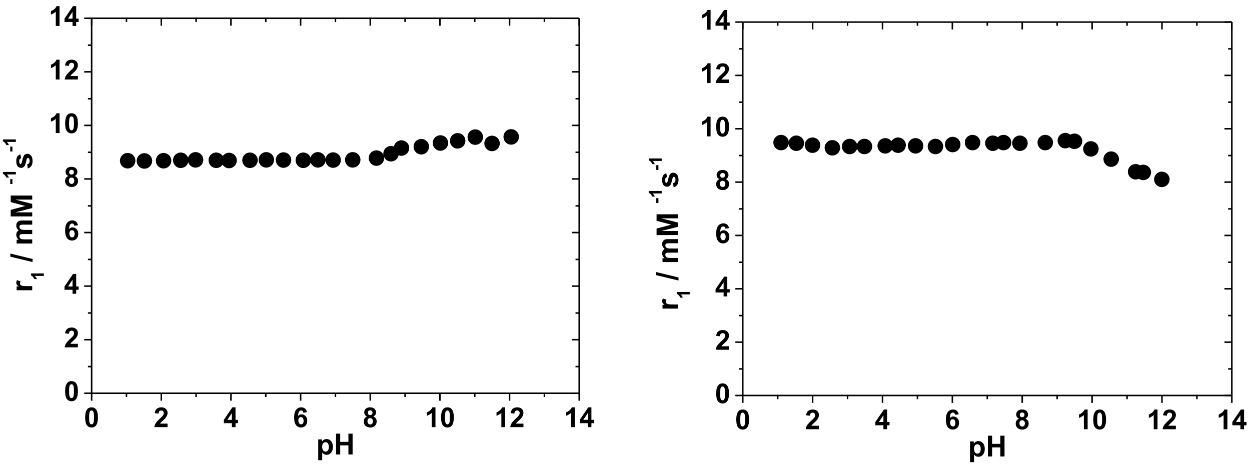


**Figure S2.** pH dependent relaxivities ($r_1$) of $Gd_2$**L1** (left) and $Gd_2$**L2** (right) measured at 20 MHz and 298 K.

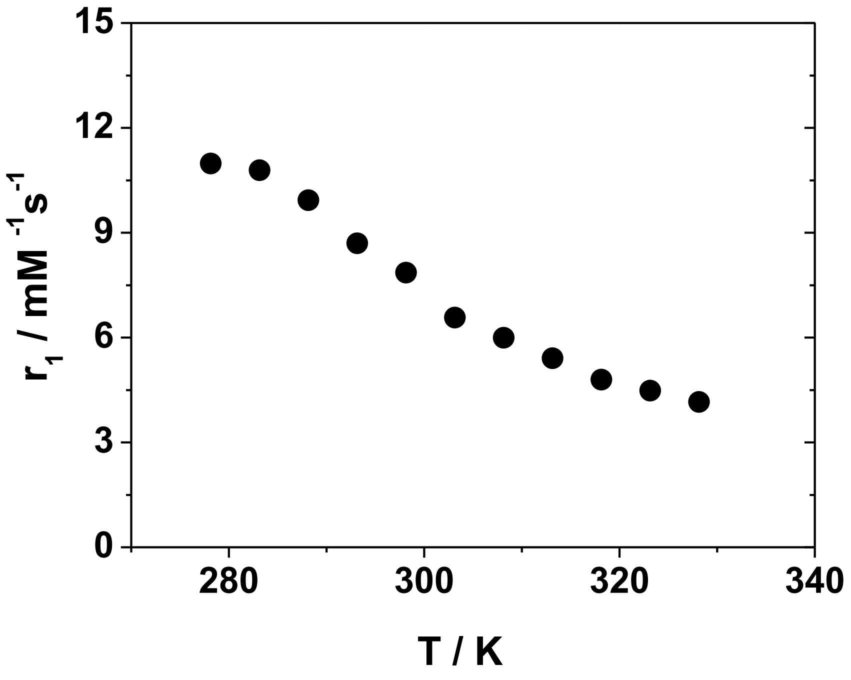


**Figure S3.** Temperature dependence of the longitudinal water proton relaxivity for $Gd_2$**L1**at 20 MHz and pH = 7.

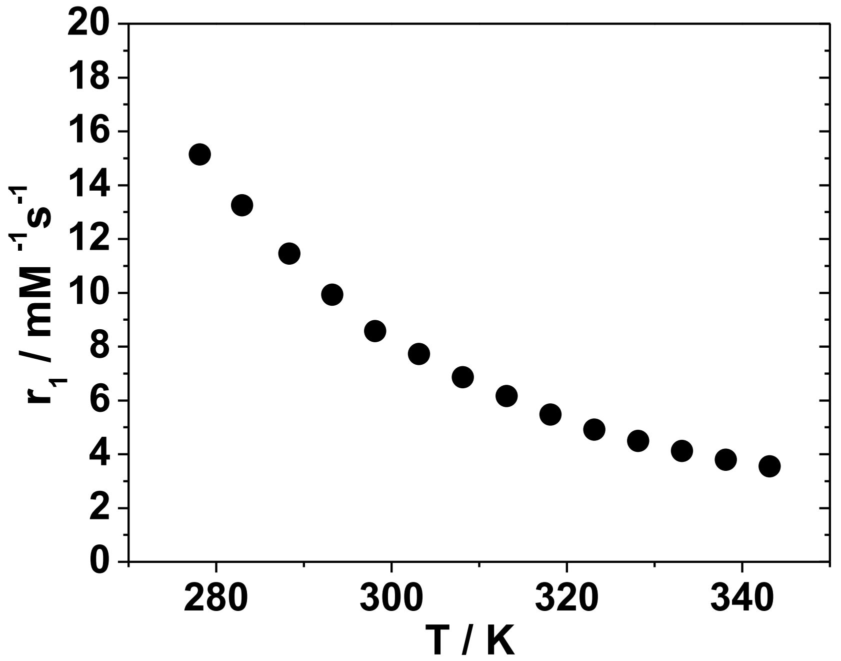


**Figure S4.** Temperature dependence of the longitudinal water proton relaxivity for $Gd_2$**L2** at 20 MHz and pH = 7.

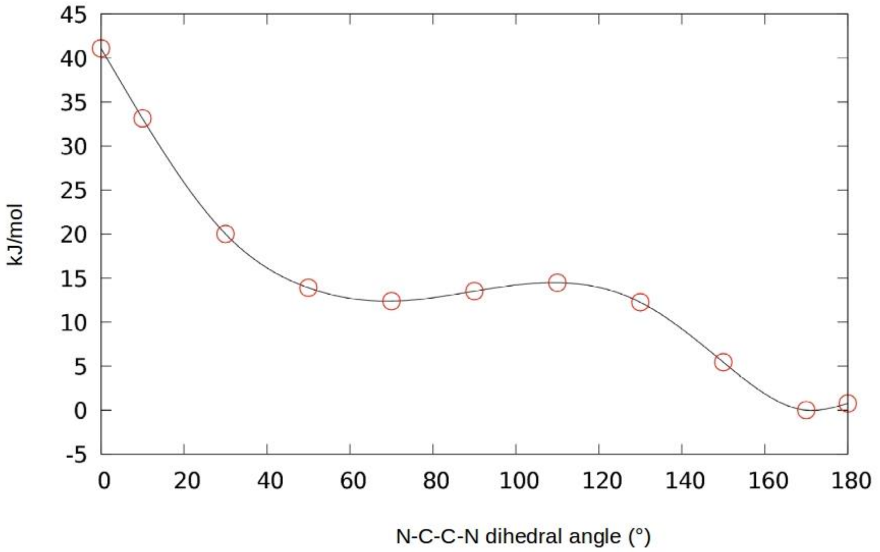


**Figure S5.** Energy scan for the rigid rotation of $Gd_2$**L2** in vacuo around the linker connecting the two $Gd^{III}$ complexes.

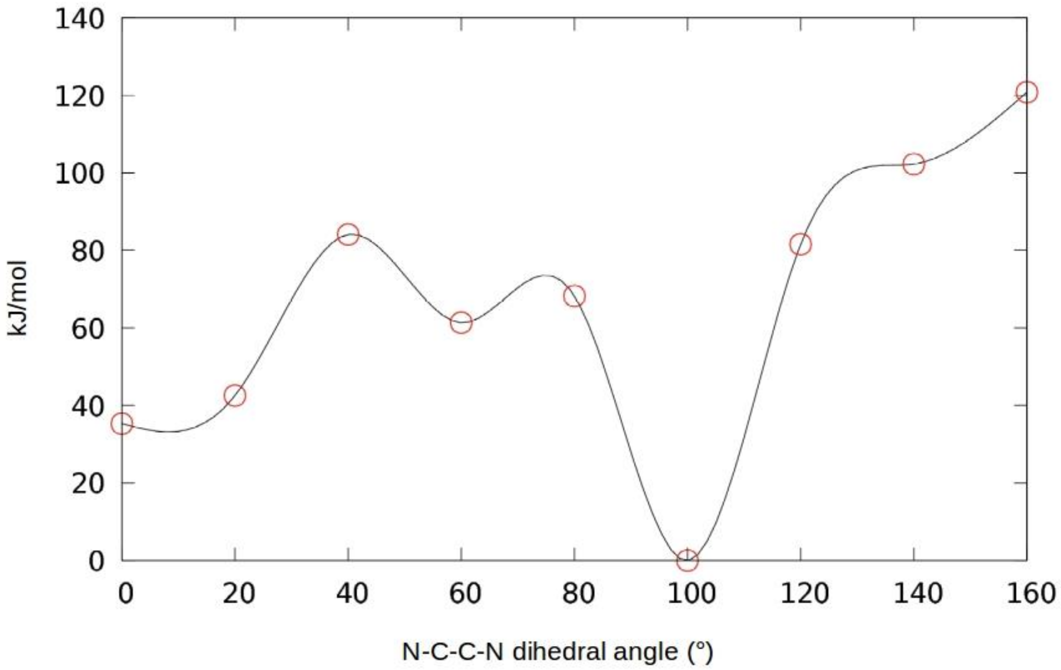


**Figure S6.** Energy scan for the rigid rotation $Gd_2$**L2** in water (9 explicit molecules + PCM for long range) around the branch connecting the two $Gd^{III}$ complexes.

### 3. $^{1}$H and $^{13}$C NMR spectra and LC-MS chromatogram

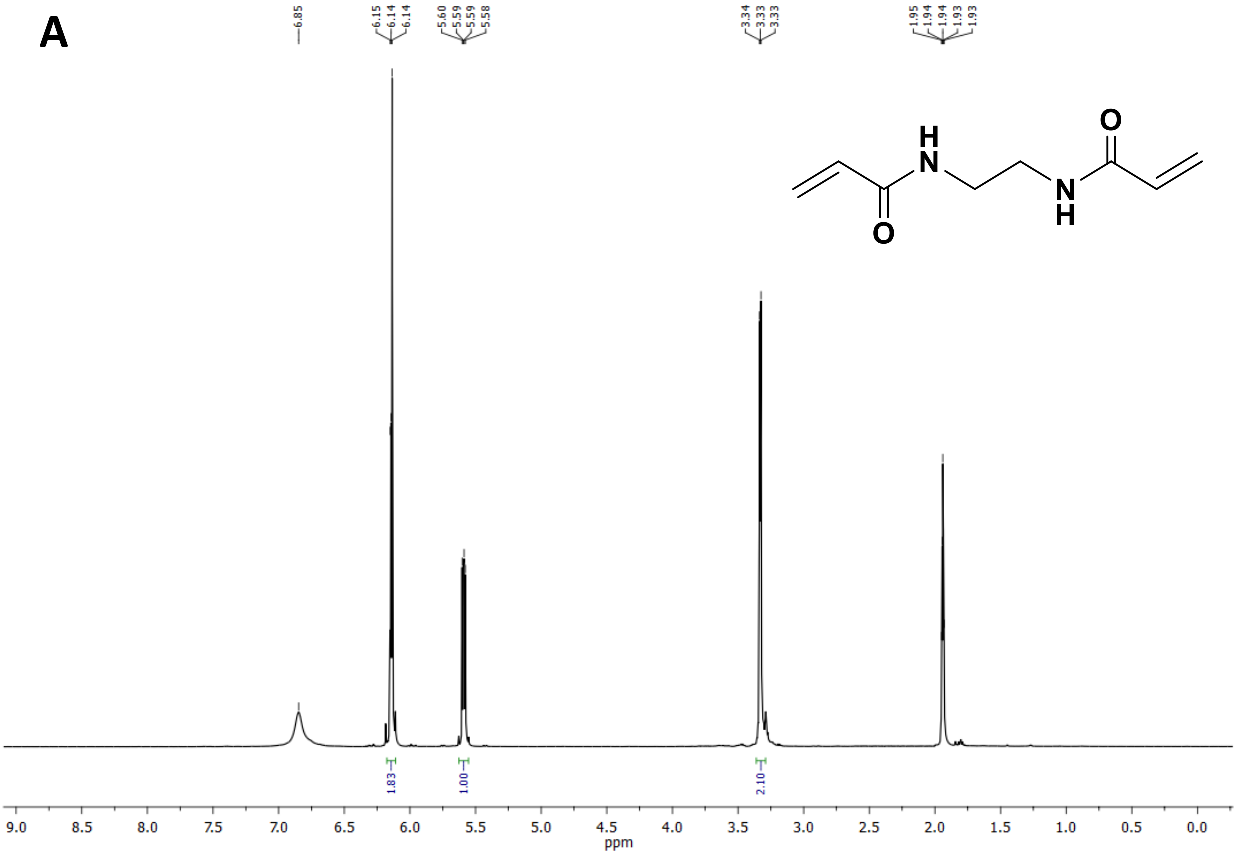


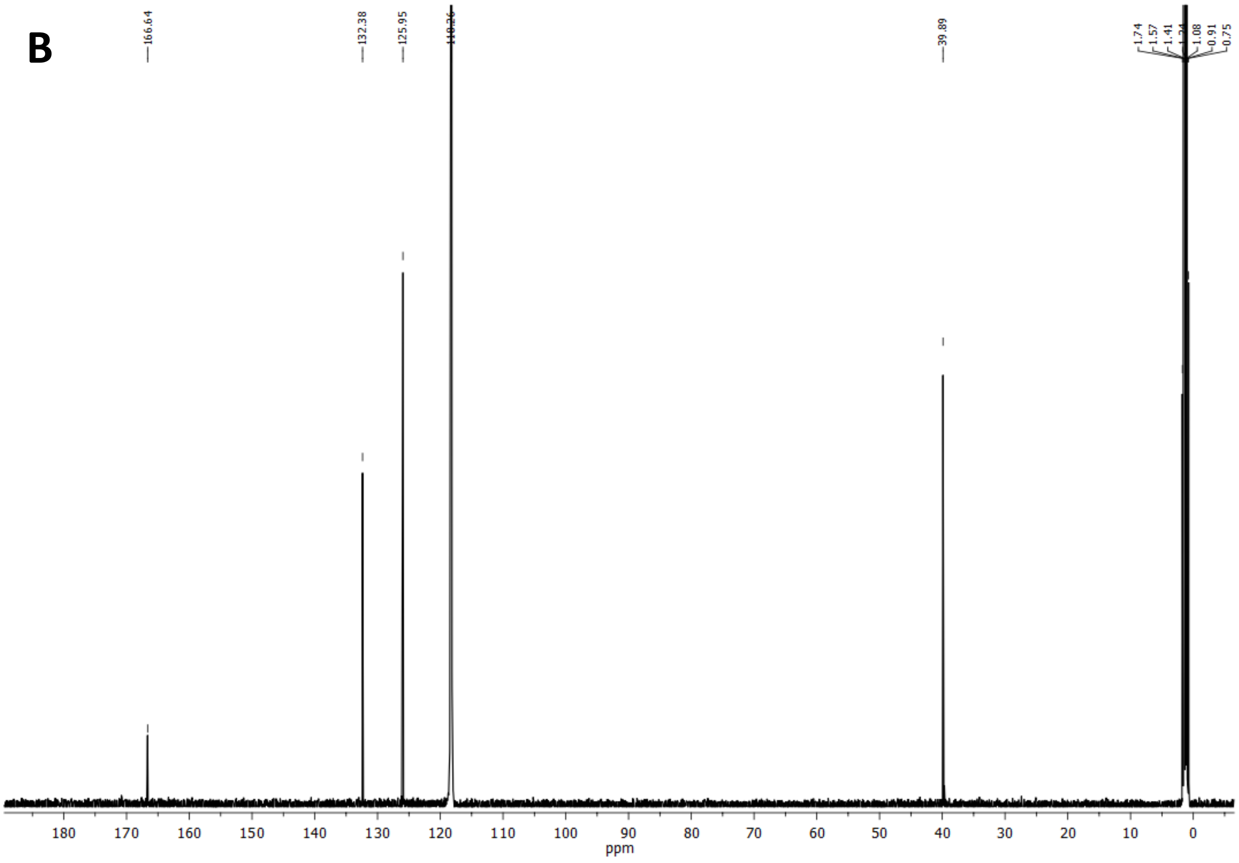

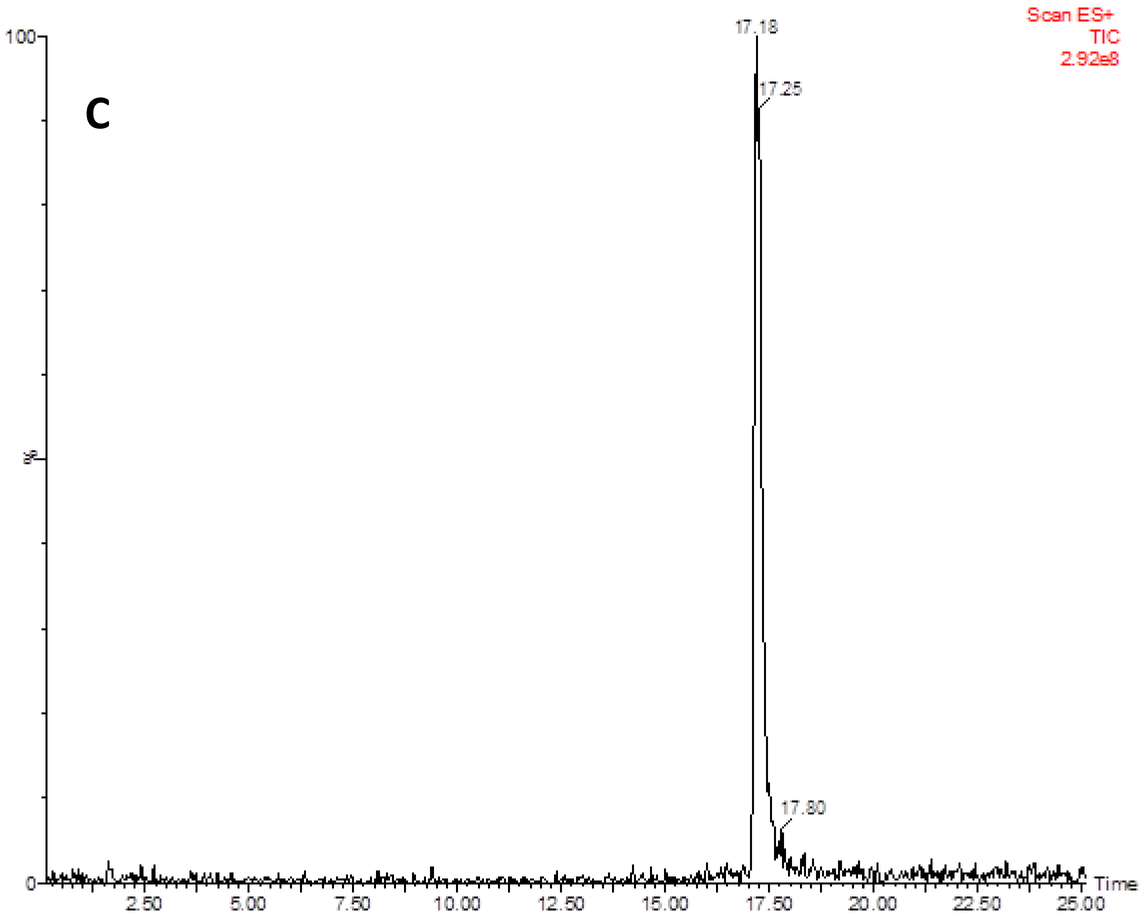


**Figure S7**. (a) $^1$H NMR, (b) $^{13}$C NMR spectra and (c) LC-MS chromatografic profile of 1,2-diacrylamidoethane

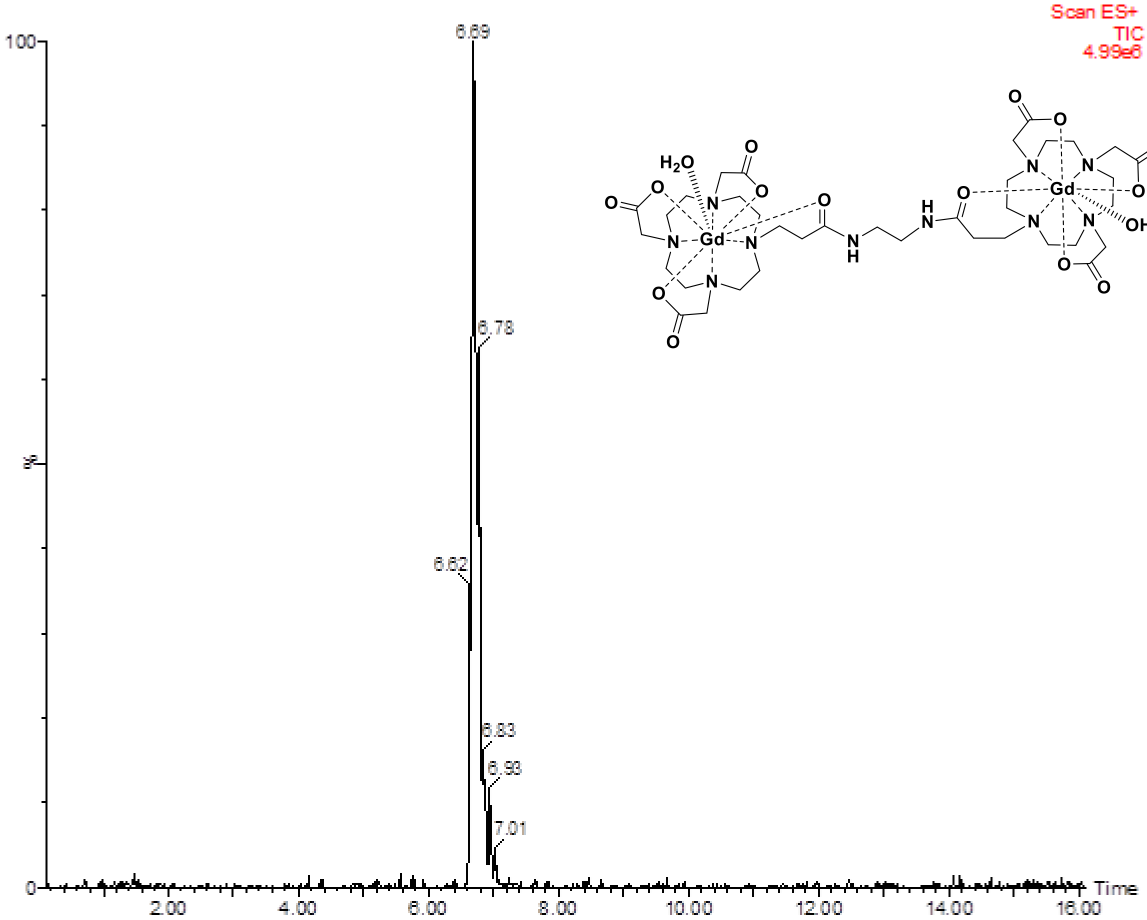


**Figure S8**. LC-MS chromatografic profile of Gd$_2$**L1**

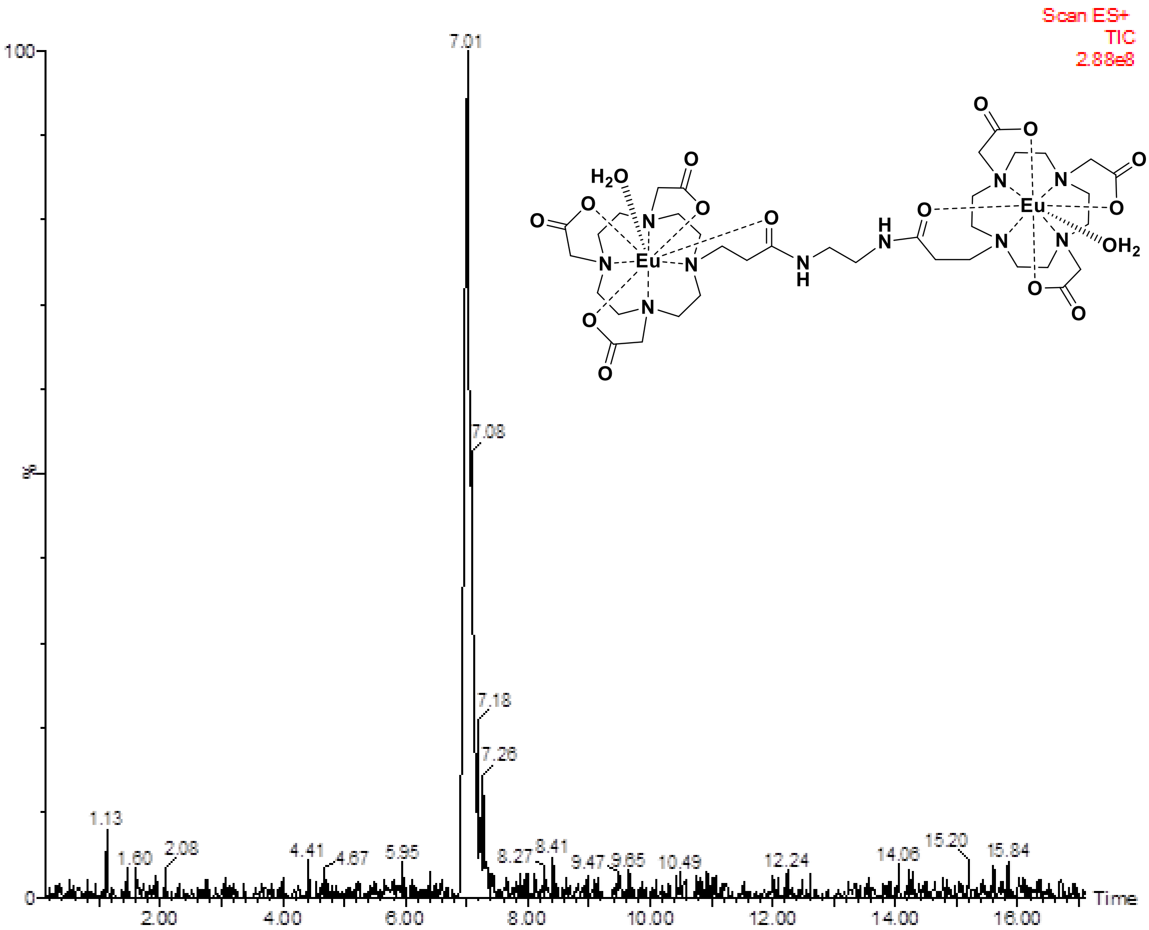


**Figure S9**. LC-MS chromatografic profile of Eu$_2$**L1**

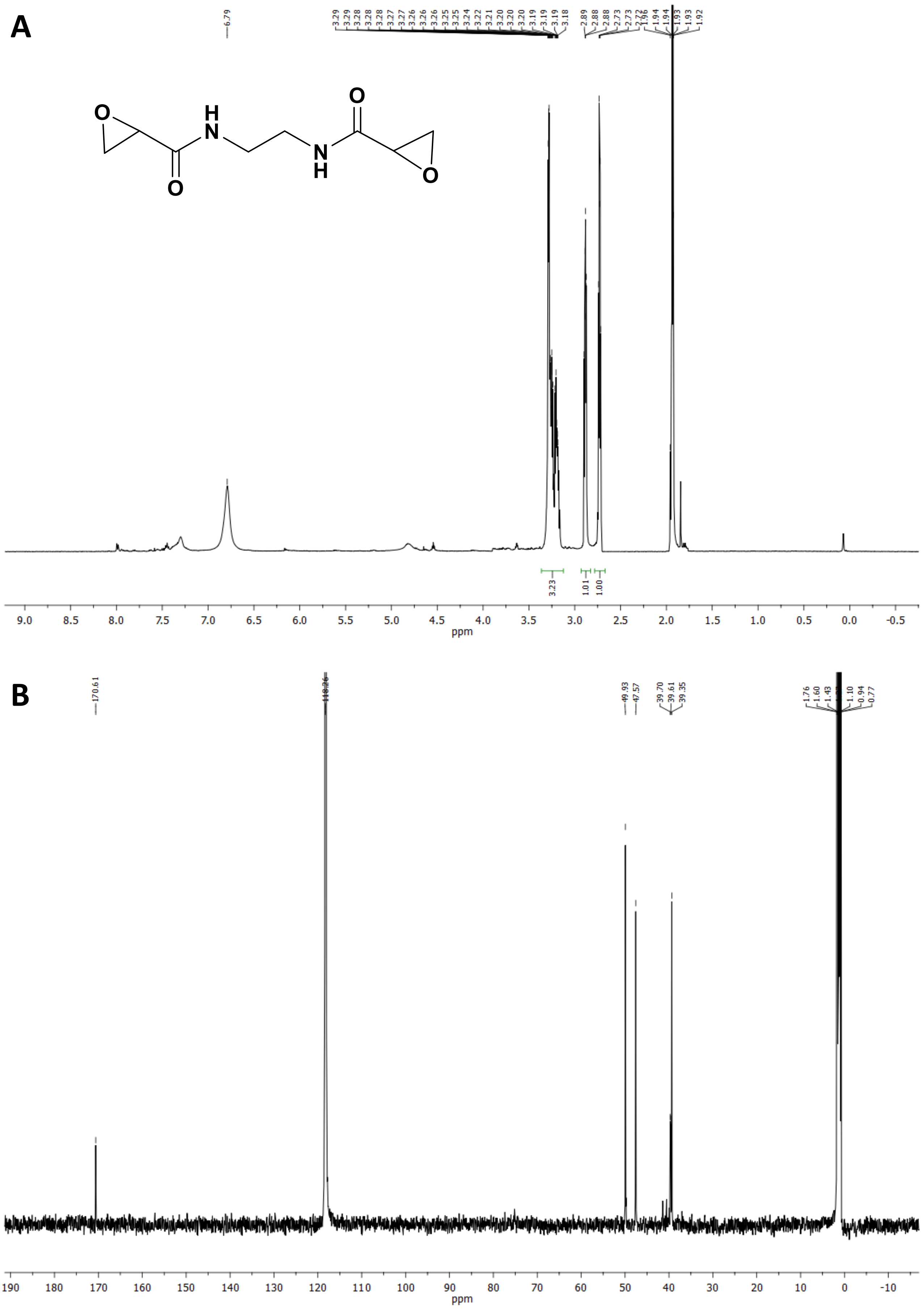
A
6.79
3.23
1.01
1.00
ppm
B
170.61
118.26
49.93
47.57
39.70
39.61
39.35
ppm

C

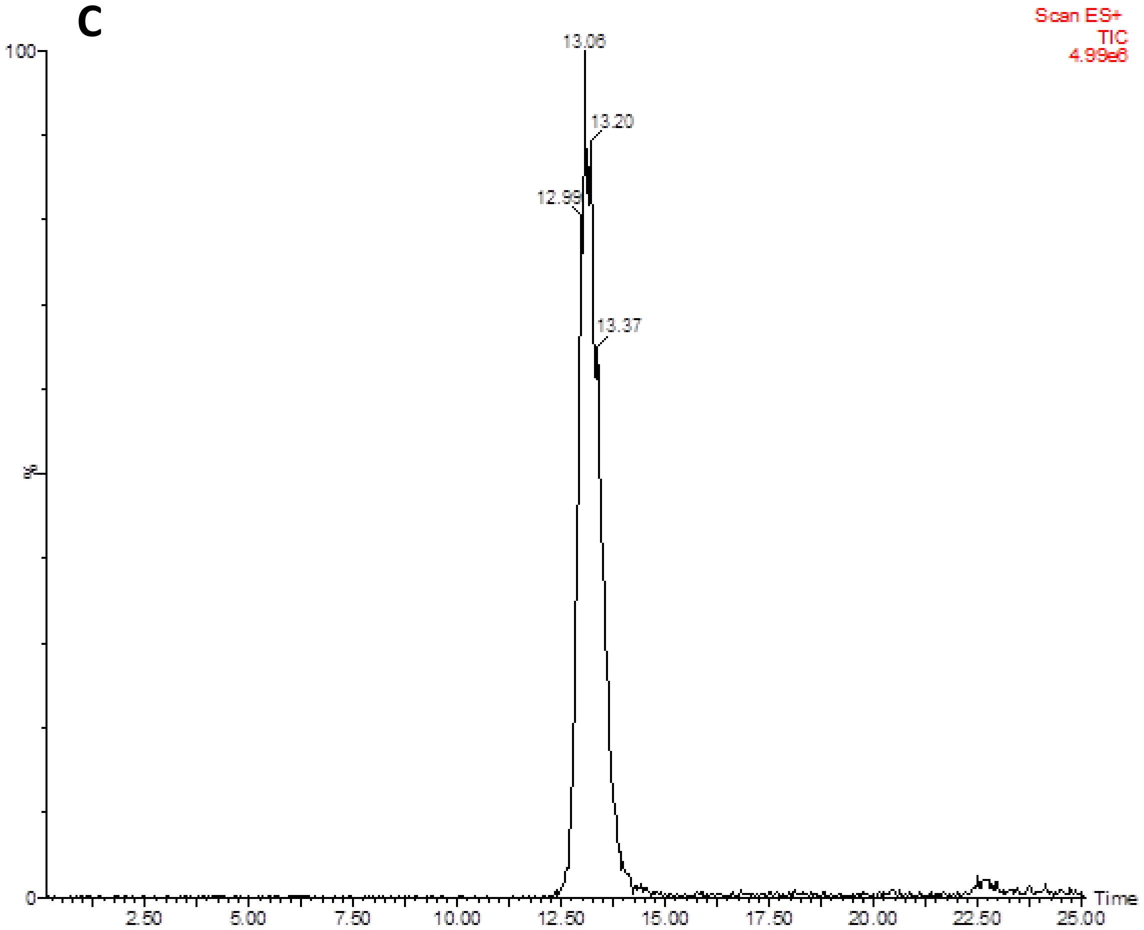


**Figure S10**. (a) $^{1}$H NMR, (b) $^{13}$C NMR Spectra and (c) LC-MS chromatografic profile of *N*,*N*'-(ethane-1,2-diyl)bis(oxirane-2-carboxamide)

**A**

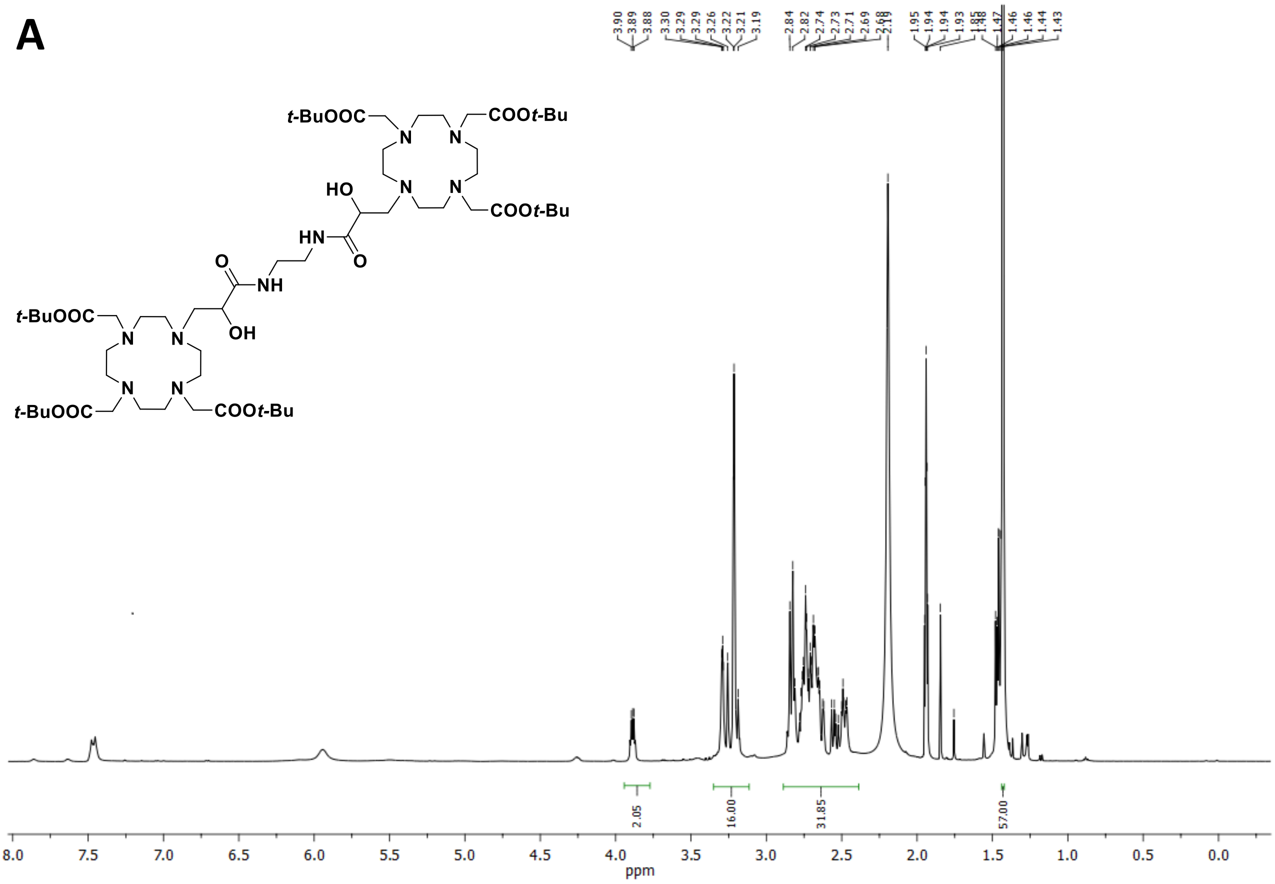

t-BuOOC
COOt-Bu
HO
HN
O
NH
OH
N
3.90 3.89 3.88 3.30 3.29 3.29 3.26 3.22 3.21 3.19 2.84 2.82 2.74 2.73 2.71 2.69 2.68 2.19 1.95 1.94 1.94 1.93 1.85 1.48 1.47 1.46 1.46 1.44 1.43
2.05
16.00
31.85
57.00
8.0 7.5 7.0 6.5 6.0 5.5 5.0 4.5 4.0 3.5 3.0 2.5 2.0 1.5 1.0 0.5 0.0
ppm


**B**

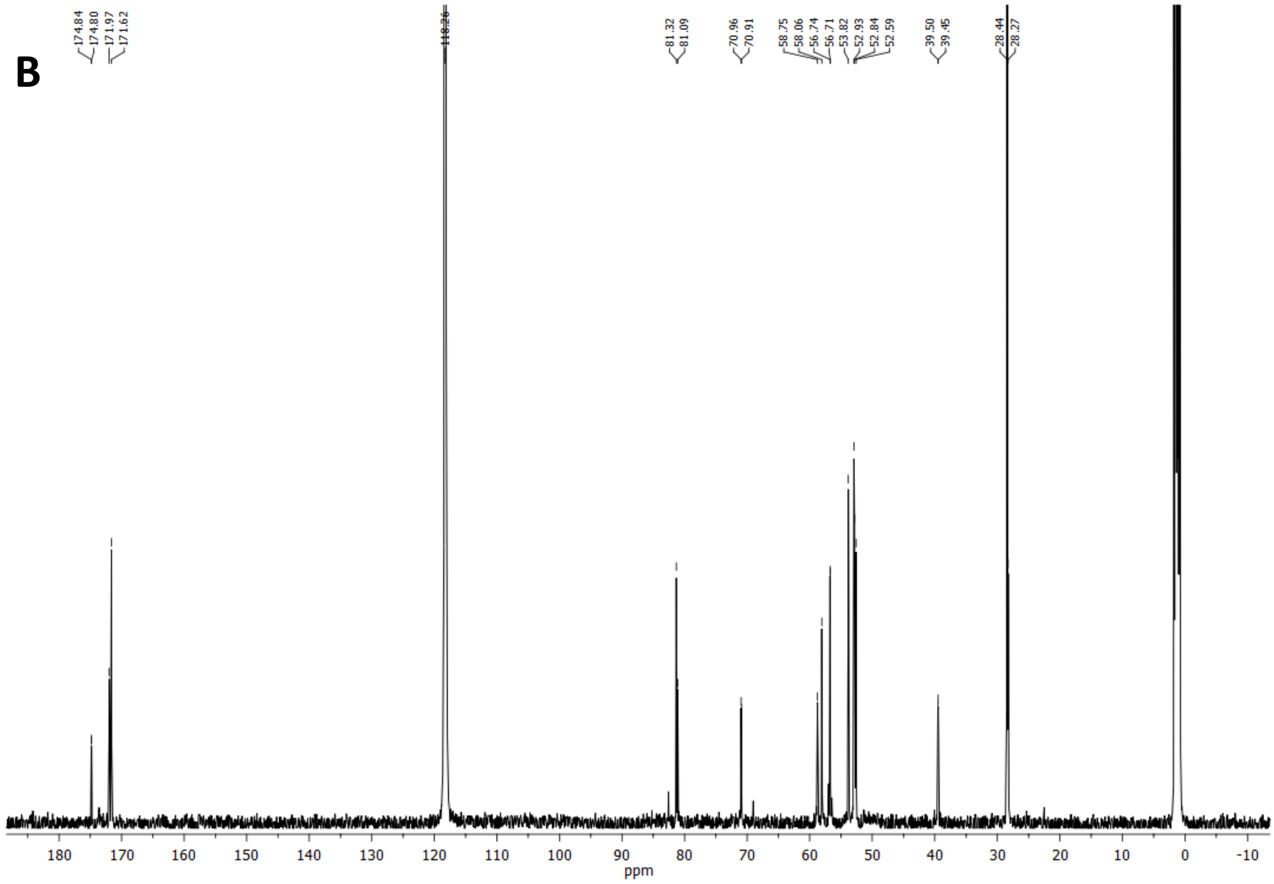

174.84 174.80 171.97 171.62
118.26
81.32 81.09
70.96 70.91
58.75 58.06 56.74 56.71 53.82 52.93 52.84 52.59
39.50 39.45
28.44 28.27
180 170 160 150 140 130 120 110 100 90 80 70 60 50 40 30 20 10 0 -10
ppm

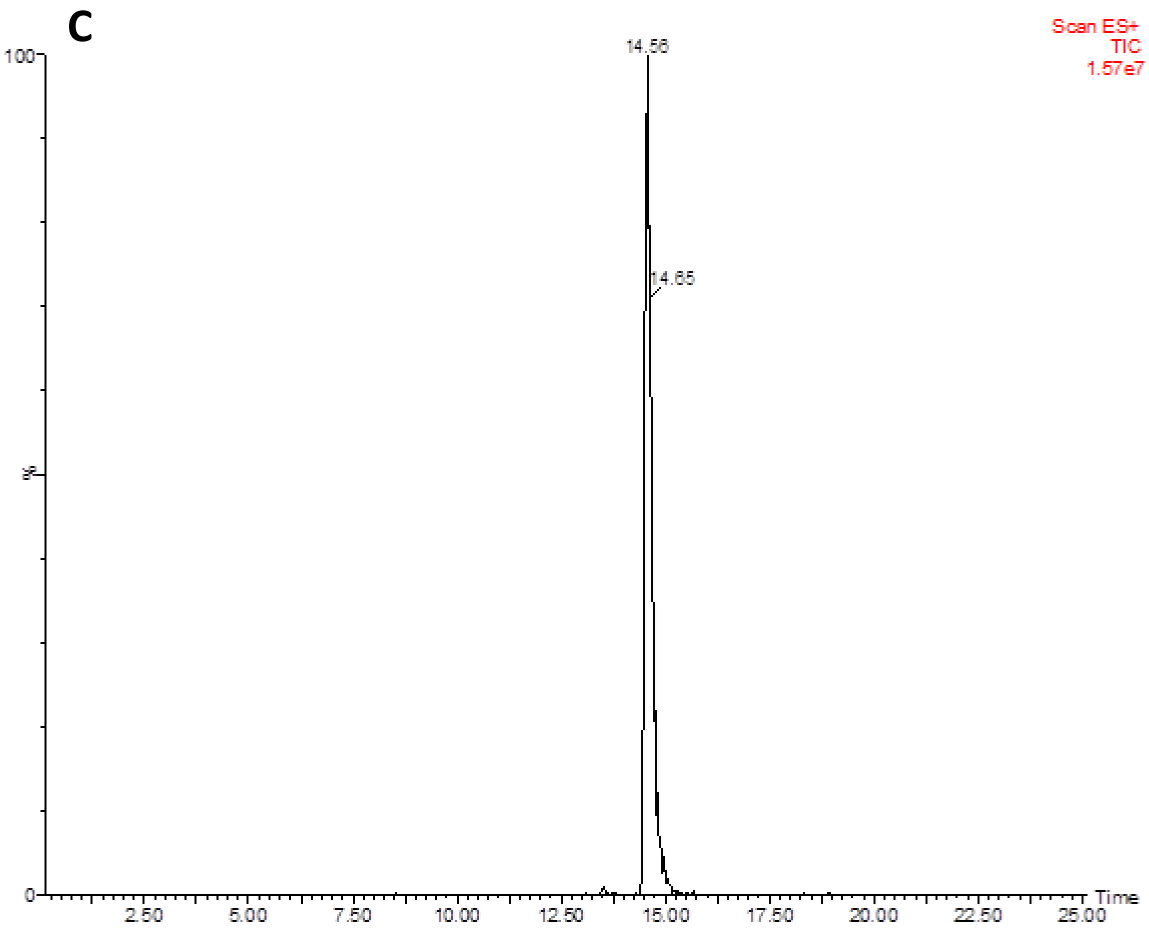


**Figure S11**. (a) $^{1}H$ NMR, (b) $^{13}C$ NMR Spectra and (c) LC-MS chromatografic profile of (HPA-DO3A)$_2$(tBu)$_6$

A

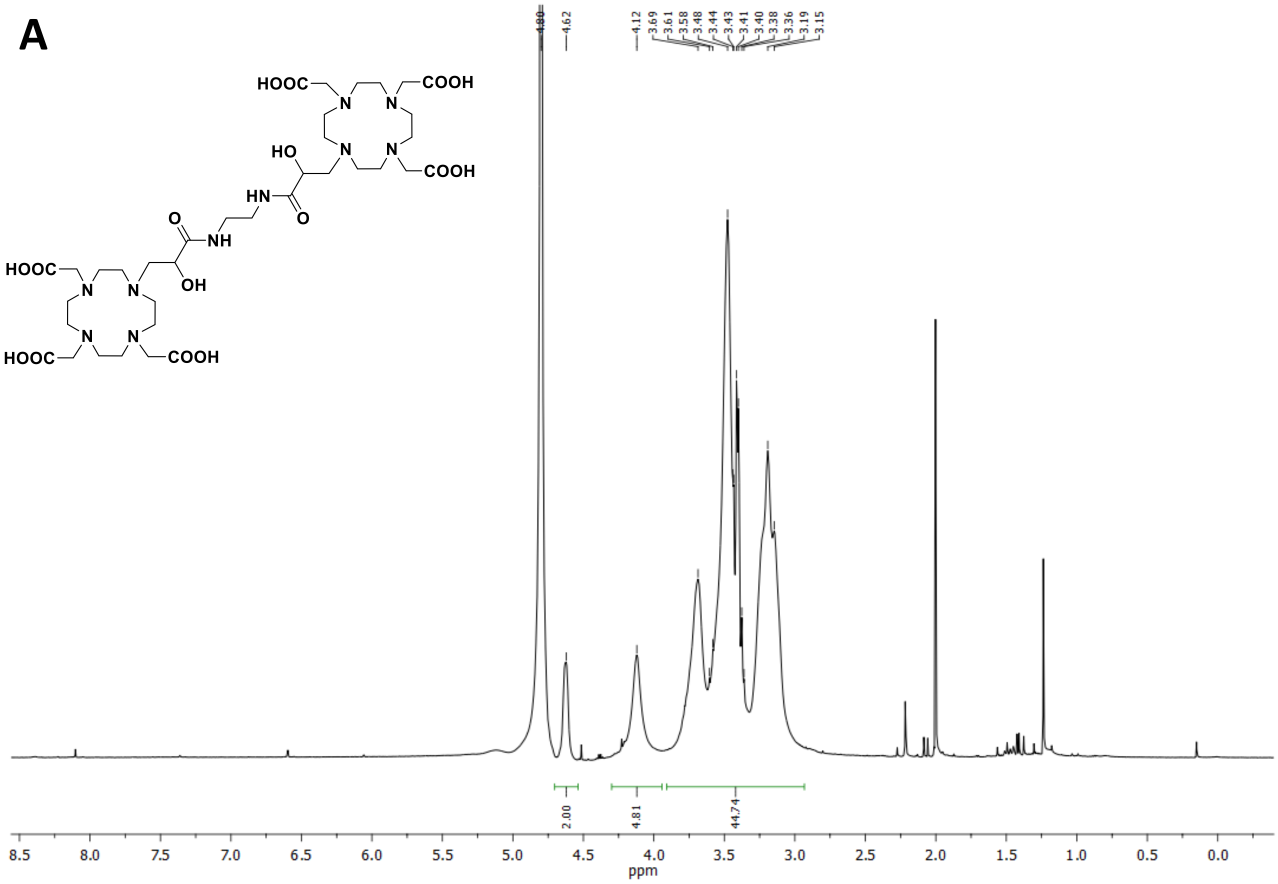
4.80
4.62
4.12
3.69
3.61
3.58
3.48
3.44
3.43
3.41
3.40
3.38
3.36
3.19
3.15
HOOC
COOH
HO
HN
O
NH
OH
2.00
4.81
44.74
8.5 8.0 7.5 7.0 6.5 6.0 5.5 5.0 4.5 4.0 3.5 3.0 2.5 2.0 1.5 1.0 0.5 0.0
ppm

B

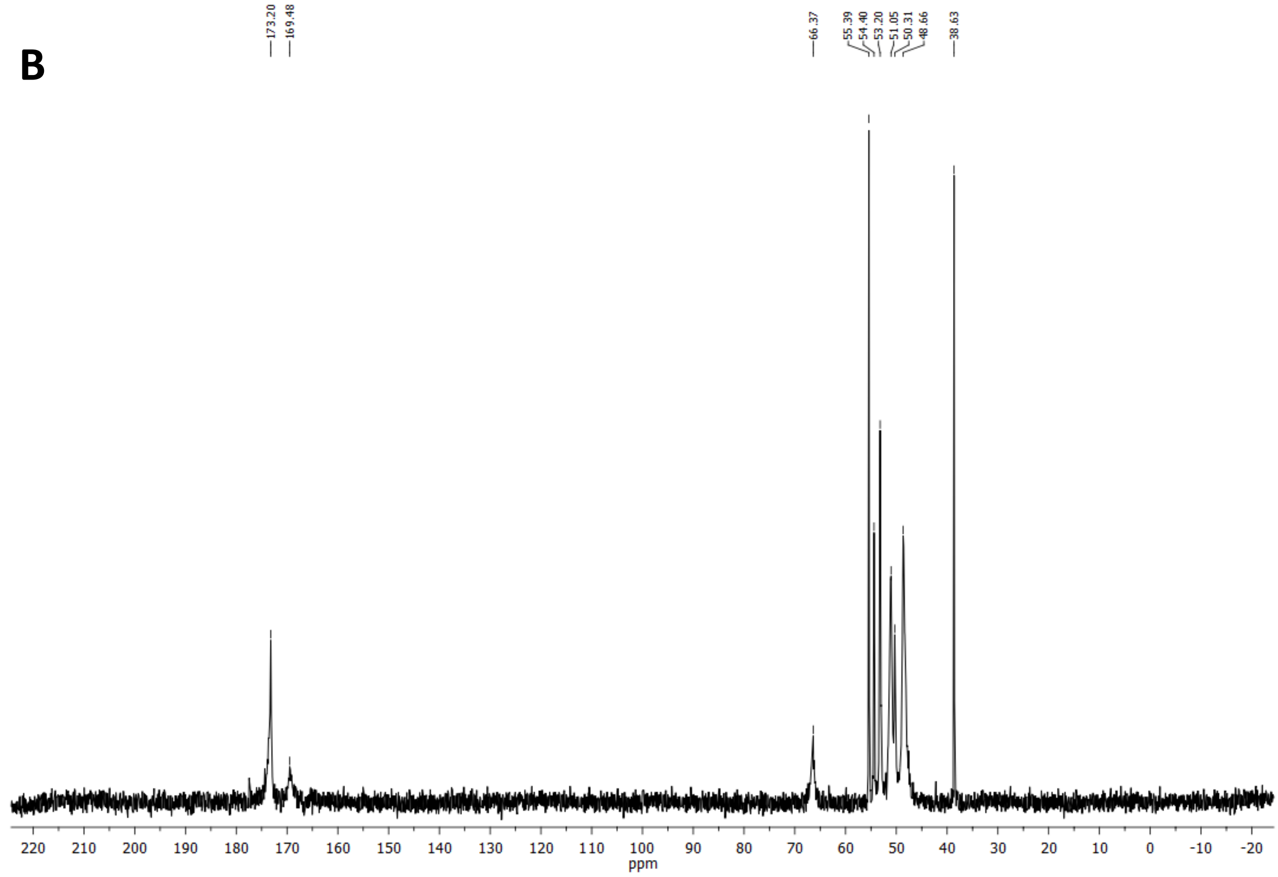
173.20
169.48
66.37
55.39
54.40
53.20
51.05
50.31
48.66
38.63
220 210 200 190 180 170 160 150 140 130 120 110 100 90 80 70 60 50 40 30 20 10 0 -10 -20
ppm

**C**

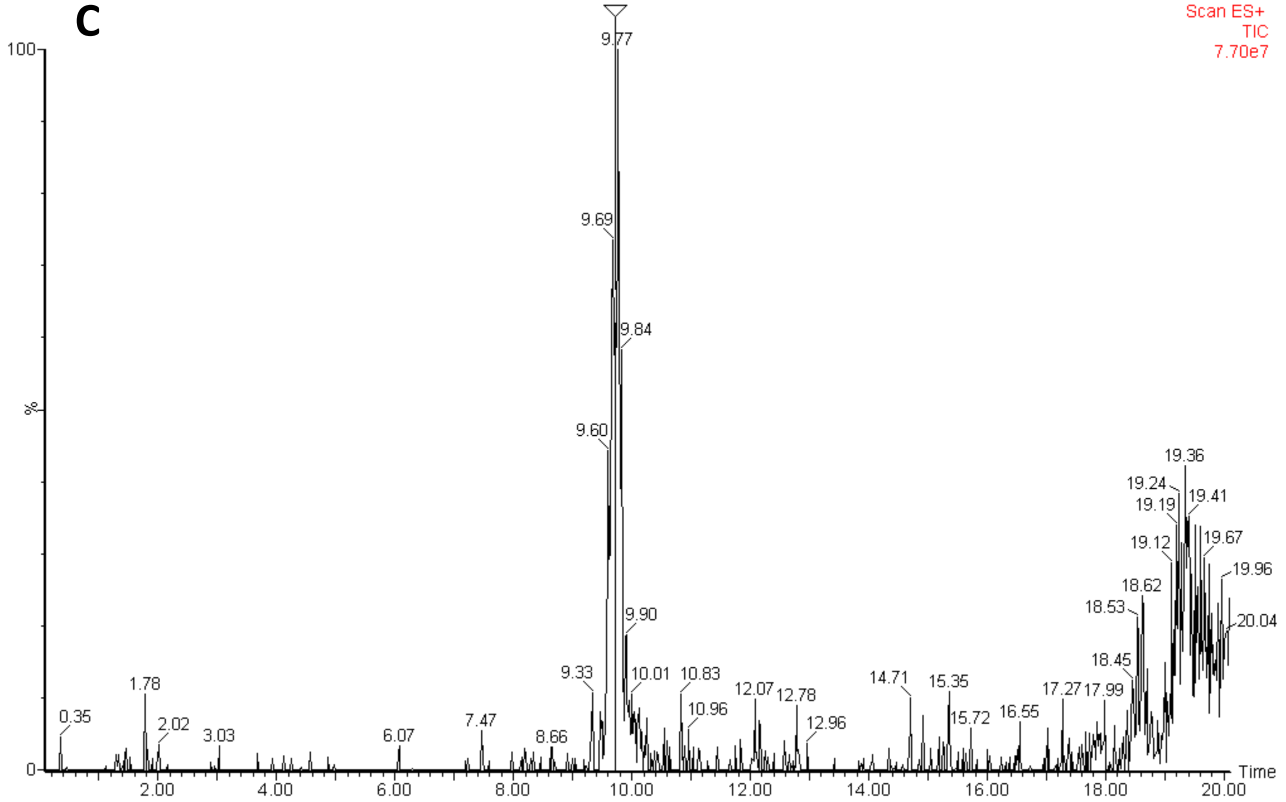


**Figure S12**. (a) $^{1}$H NMR, (b) $^{13}$C NMR spectra and (c) LC-MS chromatografic profile of (HPA-DO3A)$_2$ (**L2**)

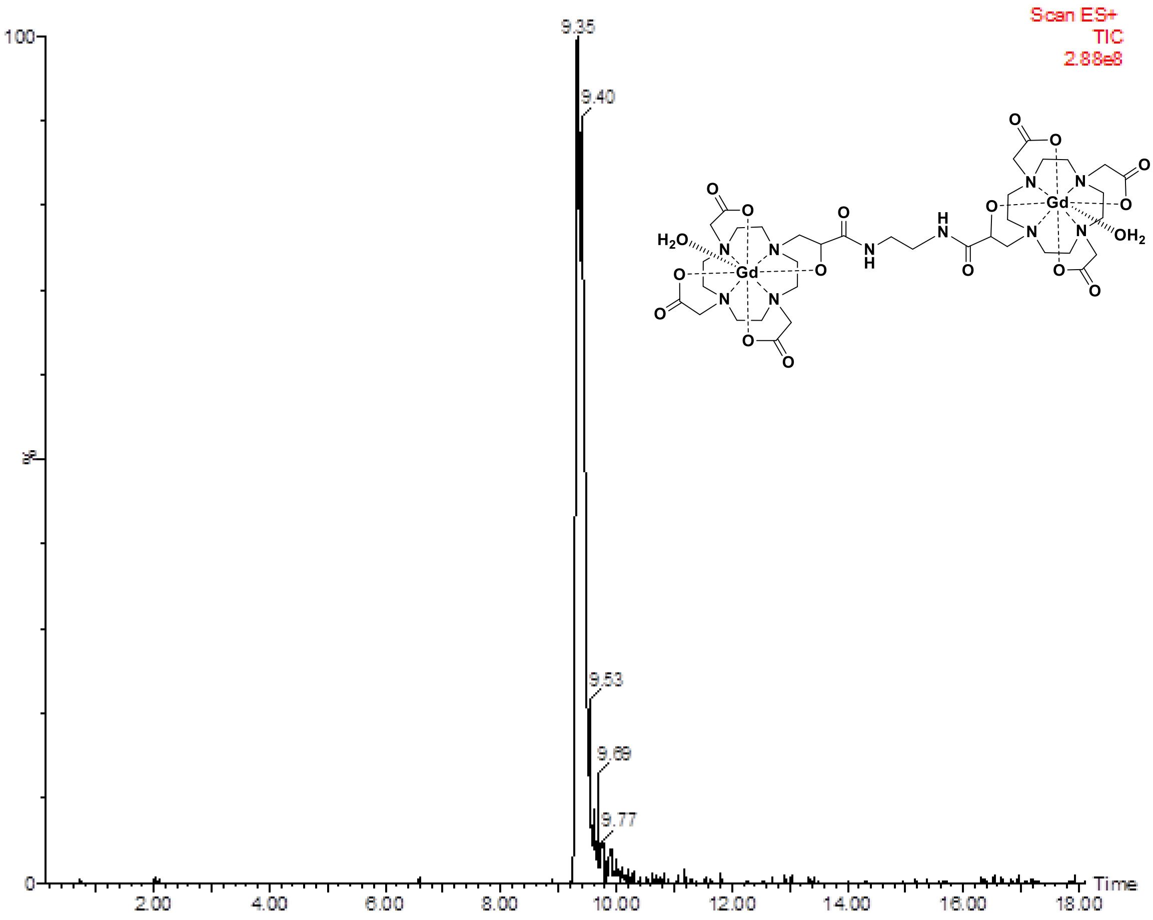


**Figure S13**. Chromatografic profile of $Gd_2$**L2**

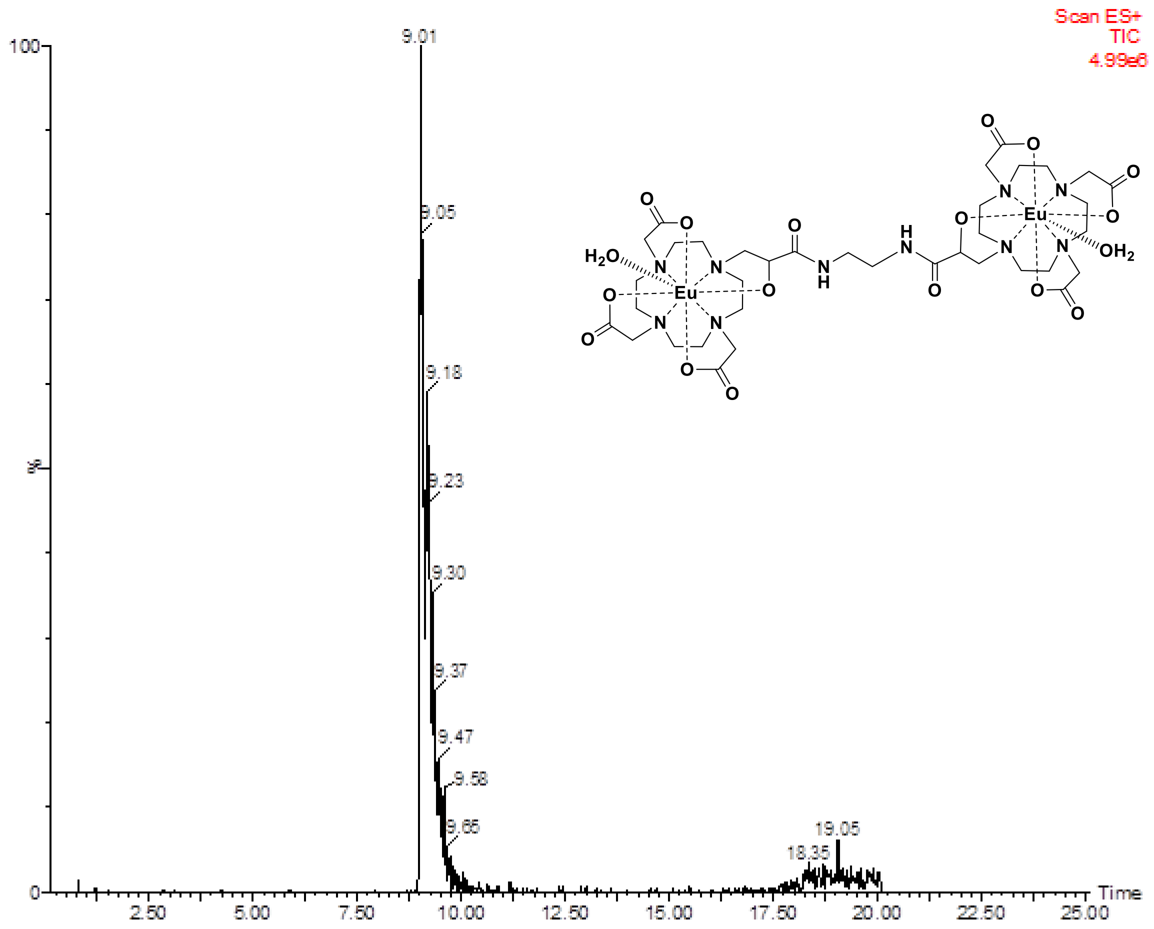


**Figure S14**. LC-MS chromatografic profile of $Eu_2$**L2**

## 4. References


(i) Swift, T. J.; Connick, R. E. *J. Chem. Phys.* **1962**, *37*, 307.

(ii) Zimmermann, J. R.; Brittin, W. E. *J. Phys. Chem.* **1957**, *61*, 1328.

(iii) McLachlan, A. D. *Proc. R. Soc. London, A*, **1964**, *280*, 271-288.

(iv) Luz, Z.; Meiboom, S. *J. Chem. Phys.* **1964**, *40*, 2686.

(v) The Chemistry of Contrast Agents in Medical Magnetic Resonance Imaging (Eds: Merbach, A. E.; Tóth, É.), Wiley, New York, **2001**.

(vi) Freed, J. H. *J. Chem. Phys.* **1978**, *68*, 4034.

(vii) Koenig, S. H.; Brown III, R. D. *Prog. Nucl. Magn. Reson. Spectrosc.* **1991**, *22*, 487.
